# Cell differentiation modifies the p53 transcriptional program through a combination of gene silencing and constitutive transactivation

**DOI:** 10.1038/s41418-023-01113-4

**Published:** 2023-01-21

**Authors:** Roubina Tatavosian, Micah G. Donovan, Matthew D. Galbraith, Huy N. Duc, Maria M. Szwarc, Molishree U. Joshi, Amy Frieman, Ganna Bilousova, Yingqiong Cao, Keith P. Smith, Kunhua Song, Angela L. Rachubinski, Zdenek Andrysik, Joaquin M. Espinosa

**Affiliations:** 1 Department of Pharmacology, School of Medicine, University of Colorado Anschutz Medical Campus, Aurora, CO 80045 USA; 2 Linda Crnic Institute for Down Syndrome, School of Medicine, University of Colorado Anschutz Medical Campus, Aurora, CO 80045 USA; 3 Functional Genomics Facility, School of Medicine, University of Colorado Anschutz Medical Campus, Aurora, CO 80045 USA; 4 Charles C. Gates Center for Regenerative Medicine, University of Colorado Anschutz Medical Campus, Aurora, CO 80045 USA; 5 Division of Cardiology, Department of Medicine, University of Colorado Anschutz Medical Campus, Aurora, CO 80045 USA; 6 Section of Developmental Pediatrics, Department of Pediatrics, University of Colorado Anschutz Medical Campus, Aurora, CO 80045 USA

**Keywords:** Tumour-suppressor proteins, Epigenetics

## Abstract

The p53 transcription factor is a master regulator of cellular responses to stress that is commonly inactivated in diverse cancer types. Despite decades of research, the mechanisms by which p53 impedes tumorigenesis across vastly different cellular contexts requires further investigation. The bulk of research has been completed using in vitro studies of cancer cell lines or in vivo studies in mouse models, but much less is known about p53 action in diverse non-transformed human tissues. Here, we investigated how different cellular states modify the p53 transcriptional program in human cells through a combination of computational analyses of publicly available large-scale datasets and in vitro studies using an isogenic system consisting of induced pluripotent stem cells (iPSCs) and two derived lineages. Analysis of publicly available mRNA expression and genetic dependency data demonstrated wide variation in terms of expression and function of a core p53 transcriptional program across various tissues and lineages. To monitor the impact of cell differentiation on the p53 transcriptome within an isogenic cell culture system, we activated p53 by pharmacological inhibition of its negative regulator MDM2. Using cell phenotyping assays and genome wide transcriptome analyses, we demonstrated that cell differentiation confines and modifies the p53 transcriptional network in a lineage-specific fashion. Although hundreds of p53 target genes are transactivated in iPSCs, only a small fraction is transactivated in each of the differentiated lineages. Mechanistic studies using small molecule inhibitors and genetic knockdowns revealed the presence of two major regulatory mechanisms contributing to this massive heterogeneity across cellular states: gene silencing by epigenetic regulatory complexes and constitutive transactivation by lineage-specific transcription factors. Altogether, these results illuminate the impact of cell differentiation on the p53 program, thus advancing our understanding of how this tumor suppressor functions in different contexts.

## Introduction

The p53 protein is a potent tumor suppressor acting as a master transcriptional regulator of the cellular response to stress. p53 represses tumor growth across human tissues by regulating expression of hundreds of target genes involved in various anti-tumoral processes, including apoptosis, cell cycle arrest, and senescence [1–6]. p53 protein levels are tightly regulated by its repressor murine double minute 2 (MDM2), which targets p53 for ubiquitin-dependent degradation and also blocks its transactivation domain [7–9]. Upon stress stimuli such as DNA damage and oncogene hyperactivation, p53 is activated by signaling pathways that disrupt the MDM2-p53 interaction, leading to p53 stabilization and post-translational modifications [10]. In the cell nucleus, p53 binds to p53 response elements (p53REs) to induce transcription of its target genes and evoke different cellular outcomes [1–3, 11, 12]. However, despite several decades of research, there is an incomplete understanding of how the p53 network functions in different cellular contexts to suppress tumors originating from diverse tissues.

p53 is inactivated in most human cancers, either via mutations impairing its DNA binding activity [13–15] or by overexpression of MDM2 or other negative regulators [16, 17]. Targeted pharmacological inhibition of the MDM2-p53 interaction is being tested in cancer therapy, with several small molecule inhibitors already developed, such as the first-in-class compound Nutlin-3 [18]. Although these molecules specifically activate p53 and its downstream target genes, they elicit highly diverse responses across cell types [4, 19]. In most cancer cell types the response to MDM2 inhibition is a reversible form of cell cycle arrest of little therapeutic value, with only few cell lines undergoing apoptosis [4]. Highly diverse p53-dependent cellular responses are also observed at the organismal level among non-transformed cell types [20–22]. For example, in mouse models harboring “switchable” p53 expression constructs, p53 induces apoptosis in the spleen, thymus, and bone marrow, and cell cycle arrest in tissues such as brain, lung, heart, liver, and kidneys [22]. These observations reveal a dearth of knowledge about the mechanisms by which p53 functions in diverse somatic tissues.

Here, we report an analysis of the impact of cell differentiation on the p53 transcriptional network using computational studies of large-scale datasets from hundreds of normal tissues and cancer cell types, and an isogenic system consisting of human iPSCs and two differentiated lineages. Our results demonstrate that cell differentiation strongly modifies the p53 transcriptional program in a lineage-specific fashion through two major mechanisms: gene silencing by epigenetic regulatory complexes and constitutive transactivation by lineage-specific transcription factors. In differentiated cell types, hundreds of potential p53 target genes become refractory to p53 action either by silencing or constitutive induction. Altogether, our results reveal the vast impact of cell differentiation on the p53 transcriptional program, supporting the notion that p53 may exert its anti-tumoral effects by different mechanisms in diverse cellular contexts.

## Results

### Functional heterogeneity in the p53 transcriptional program across human cell types

**Fig. 1:**
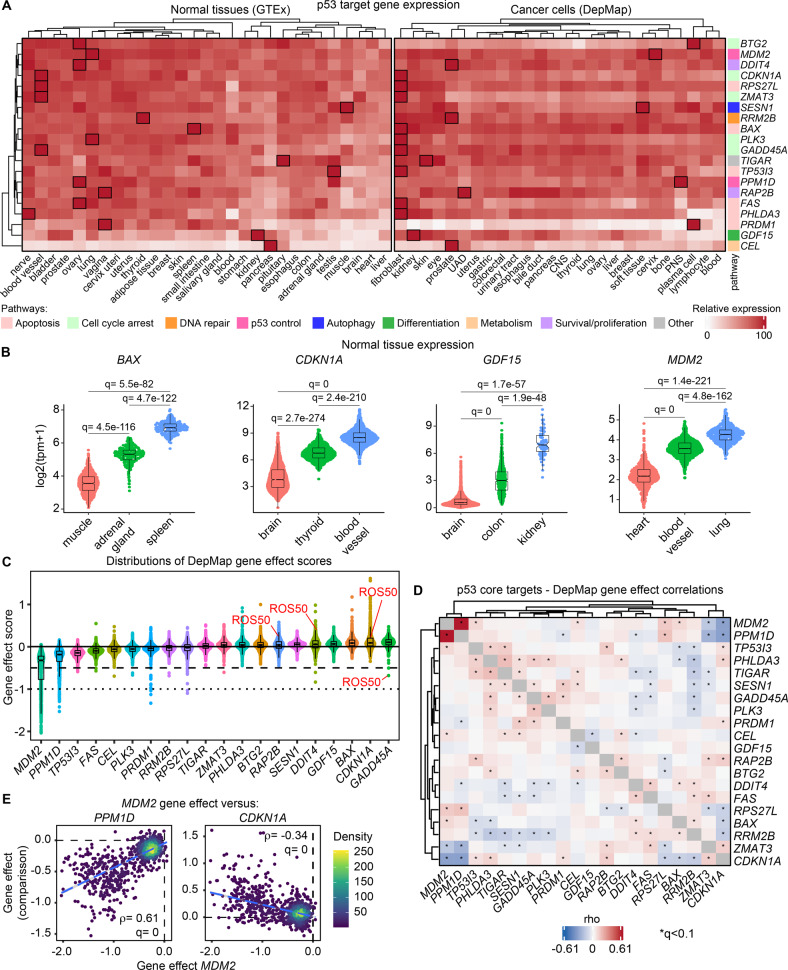
The p53 transcriptional program displays strong heterogeneity in expression and function across human cell types. **A** Relative expression of a select panel of direct p53 target genes in normal/healthy tissues (left) and cancer cell types (right). For both normal tissues and cancer cells, expression of each gene was normalized relative to the tissue or cancer cell lineage with its highest expression (indicated with black boxes). Rows were clustered based on relative expressions in normal tissues. The Genotype-Tissue Expression (GTEx) dataset employed here contains expression data from 29 non-diseased (normal) tissues from ~980 individuals comprising 17,373 total samples, whereas the data employed here from the Cancer Dependency Map (DepMap) project comprises expression data from 1374 cancer cell lines encompassing 26 distinct lineages. **B** Sina plots showing distribution of expression for *BAX*, *CDKN1A*, *GDF15*, and *MDM2* in tissues with the lowest (red), median (green), and highest (blue) expression. TPM (Transcript per Million) values were adjusted by log_2_(TPM + 1). Sample sizes are as follows: muscle (*n* = 803), adrenal gland (*n* = 258), spleen (*n* = 241), brain (*n* = 2642), blood vessel (*n* = 1335), colon, (*n* = 779), kidney (*n* = 89), heart (*n* = 861), lung (*n* = 578). Significance was defined by Wilcox test with Benjamini Hochberg method for multiple hypothesis correction. Boxes indicate the median and interquartile ranges. **C** Sina plots displaying distributions of gene effect scores derived from the DepMap project for a select panel of p53 target genes across 1070 cancer cell lines. Dashed line (gene effect score = -0.5) represents essentiality for cell viability. Dotted line (gene effect score = −1.0) represents strong killing effect upon gene knock-out. Sample size is 1070. Boxes indicate the median and interquartile ranges. **D** Heatmap showing Spearman correlation matrix of DepMap gene effect scores from 1070 cancer cell lines for a select panel of p53 target genes. Asterisks indicate significance by Spearman association test after multiple hypothesis correction using Benjamini Hochberg method (*q* < 0.1, FDR < 10%). **E** Scatter plots showing DepMap gene effect scores for *MDM2* compared to *PPM1D* (left) and *CDKN1A* (right) across 1070 cancer cell lines. *q* and rho values from Spearman association test after multiple hypothesis correction using Benjamini Hochberg method See also Figure S1 and Supplemental Files 1 and 2.

To investigate the impact of different cellular states on the p53 network, we first analyzed gene expression data obtained from hundreds of human normal tissues and cancer cell lines. We focused on a set of high-confidence 103 direct p53 target genes, referred to as “core p53 targets”, which are induced upon MDM2 inhibition at the nascent RNA level in a p53-dependent manner across multiple cancer cell types [4]. The Genotype-Tissue Expression (GTEx) dataset contains expression data from 54 normal tissues from ~1000 individuals, whereas the Cancer Dependency Map (DepMap) project contains expression data from 1393 cancer cell lines encompassing 38 distinct lineages. In both normal tissues and cancer cell lines, most p53 target genes display highly variable basal expression levels across different lineages (see Fig. 1A for 20 representative genes and Supplemental File 1 for complete analysis). For example, in normal tissues, the pro-apoptotic gene *BAX* is expressed at its lowest level in the muscle and highest in the spleen, with the adrenal glands showing intermediate levels of expression (Fig. 1B). Such variability is also observed for *CDKN1A* (encoding the cell cycle inhibitor p21) across brain, thyroid, and blood vessels; *GDF15*, a regulator of growth and differentiation [23], across brain, colon and kidney; and *MDM2* across heart, blood vessels, and lung (Fig. 1B). Strong heterogeneity in p53 target gene expression is also observed in cancer cell lines (Fig. S1A). For example, *BAX* expression among cancer cells is lowest in upper aerodigestive lineages (UAD) and highest in fibroblast lineages; *CDKN1A* is lowest in blood and highest in fibroblasts; *GDF15* is lowest in lymphocytes and highest in kidney; and *MDM2* is lowest in thyroid lineages and highest in cervix (Fig. S1A). Additionally, whereas most p53 core target genes [4] are expressed at variable levels across a range of lineages, some targets are distinctly expressed in selected lineages. For example, *CEL*, which is involved in metabolism of dietary fat, cholesteryl esters and fat-soluble vitamins [24], is selectively overexpressed in the pancreas compared to all other normal tissues (Fig. S1B) and the pro-apoptotic gene *PRDM1* is overexpressed in plasma cancer cell lineages compared to other cancer types (Fig. S1C). Notably, these analyses can not determine whether these differences in p53 targe gene expression are driven by variations in p53 function, activity of other coregulators affecting their basal expression, or both.

Next, we investigated the functional specialization of core p53 target genes by analyzing genetic dependency data generated via genome-wide knockout screens across 1070 cancer cell lines in the DepMap project (Fig. S1D, Supplemental File 2). In this dataset, negative gene effect scores are observed upon knockout of genes required for cancer cell viability, such as prominent oncogenes, with essential genes being defined as those with a gene effect score < −0.5. On the other hand, positive gene effect scores indicate genes with negative effects on cellular proliferation and survival, such as tumor suppressor genes. This analysis demonstrates a wide range of effects on cancer cell viability among p53 target genes. Reassuringly, for *MDM2*, a negative regulator of p53, the gene effect scores are negative across all cell lines and considered essential in most of them (Fig. 1C). In contrast, for *CDKN1A*, a well characterized inhibitor of cell cycle progression, the gene effect scores are positive for most cancer cell lines. Interestingly, while some p53 targets show a mostly tumor suppressive or oncogenic distribution of gene effect scores, several genes display a wide range of effects on cancer cell fitness (Fig. 1C, Fig. S1D). For example, *DDIT4*, a factor involved in regulating cell survival and proliferation [25], is essential for the survival of some cancer cell lines but may be tumor suppressive in others (Fig. 1C). This analysis also demonstrated that different target genes have different effects on the fitness of the same cancer cell line. For example, the cell cycle regulator *GADD45A* is essential in ROS50 cells, however, knockout of several other p53 target genes (e.g., *CDNK1A*, *DDIT4*, *RAP2B*) results in positive outgrowth of this cell line (Fig. 1C). To investigate the functional similarities and differences across p53 core target genes in terms of their contributions to cancer cell fitness, we visualized their genetic co-dependencies by performing Spearman correlations of their gene effect scores across all cell lines (Fig. 1D, Supplemental File 2). Notably, the strongest co-dependent relationship among p53 target genes is between *PPM1D* and *MDM2* (Fig. 1E), both of which are negative regulators of p53 function and essential in most cancer cell lines. Reassuringly, there is also an inverse relationship between the knockout effect of *MDM2* and *CDKN1A* (Fig. 1D, E). Several key p53 target genes with known anti-tumoral activities show inverse co-dependencies, as illustrated by *CDKN1A* and *BAX* (Fig. S1E). In contrast, *CDKN1A* has positive co-dependency with *PHLDA3*, another factor involved in the intrinsic apoptotic pathway [26] and *RAP2B*, which is implicated in proliferation and migration of cancer cells [27]. Another example is *TIGAR* [28], a p53 target gene involved in regulation of glycolysis, which has significant co-dependencies with the p53 targe genes *PHLAD3* and *TP53I3*, but significant inverse dependencies with the death receptor *FAS* (Fig. S1F).

Altogether, these results demonstrate high diversity in the p53 transcriptional program in terms of both mRNA expression and cellular function as defined by the effects of gene knockout.

### Cell differentiation restrains and modifies the p53 transcriptional program

**Fig. 2:**
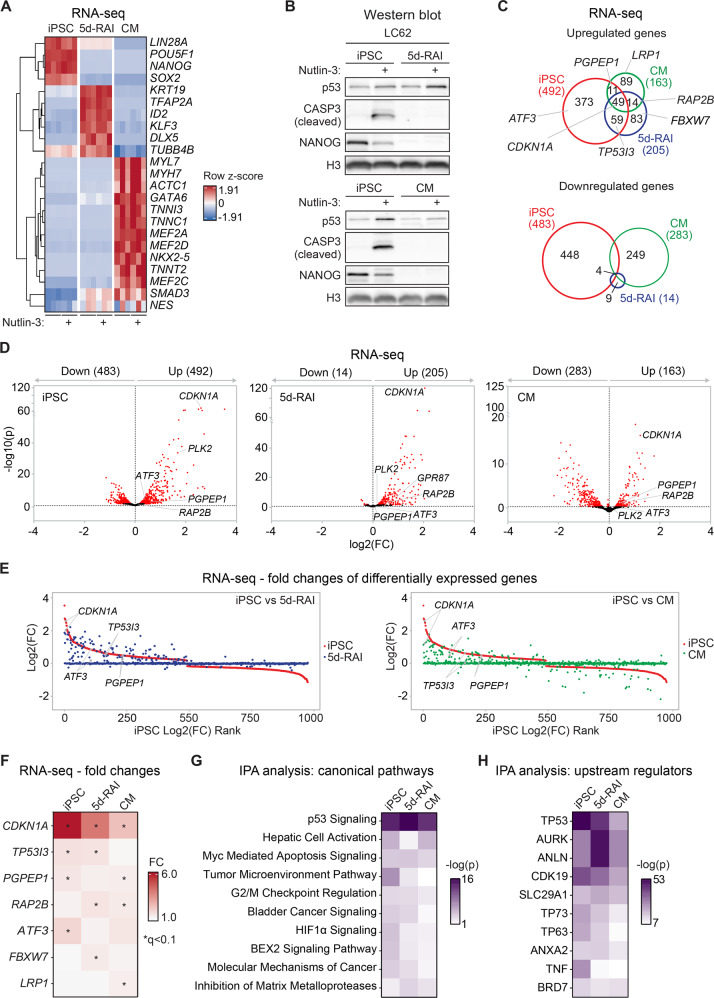
Cell differentiation modifies the p53 transcriptional program in a lineage-specific manner. **A** Heatmap displaying the absolute expression levels (in reads per kilobase per million, RPKM) for cell type-specific markers confirms the differentiation of iPSCs into RA-differentiated cells (5d-RAIs) and cardiomyocytes (CMs) with or without Nutlin-3 treatment. Row-level Z-scores of RPKMs are shown for each gene. Results shown are from three different cell lines representing independent biological replicates. **B** Immunoblots of cell line LC62 grown as iPSCs versus 5d-RAIs or CMs showing protein levels of p53, cleaved CASP3, the pluripotency marker NANOG, and the loading control histone H3. Biological replicates using two other cell lines are shown in Figure S2. **C** Venn diagrams showing overlap among statistically significant differentially expressed genes (DEGs), both downregulated and upregulated upon Nutlin-3 treatment, in the three cellular states. Indicated genes are examples of p53 core direct target genes induced in each cellular state. Statistical significance was defined with DESeq2 with a statistical cut-off of *q* < 0.1. Results shown are from three different cell lines representing independent biological replicates. **D** Volcano plots of RNA-seq results highlighting DEGs induced upon Nutlin-3 treatment in iPSCs, 5d-RAIs, and CMs (DESeq2, *q* < 0.1, labelled in red). Results shown are from three different cell lines representing independent biological replicates. **E** Rank plots of fold changes of DEGs in iPSCs compared to their fold changes in 5d-RAIs (left panel), or CMs (right panel). Results shown are from three different cell lines representing independent biological replicates. **F** Heatmap displaying the fold changes of select p53 core target genes in the three cellular states. An asterisk (*) denotes a statistically significant change (DESeq2, *q *< 0.1). Results shown are from three different cell lines representing independent biological replicates. Ingenuity Pathway Analysis (IPA) of canonical pathways (**G**) and upstream regulators (**H**) for DEGs detected in each of the three cellular states. p values are calculated by IPA using a hypergeometric test. See also Figure S2 and Supplemental Files 3 and 4.

To investigate how cellular differentiation impacts the p53 transcriptional program we employed an experimental paradigm whereby human iPSCs were differentiated into two alternative fates. The differentiation protocols involved either all-*trans* retinoic acid (RA) treatment for five days (5d-RAI) or a 45-day treatment with agents that would induce differentiation into cardiomyocytes (CM) (Fig. 2A, see Methods). We then assessed the impacts of p53 activation in each context by treating iPSCs, 5d-RAIs, and CMs with Nutlin-3, a highly specific inhibitor of the p53-MDM2 interaction [4, 18, 28, 29], or vehicle (DMSO), for 12 h. We then analyzed gene expression changes through polyA^+^ RNA-seq in three iPSC lines from different donors (labeled LC42, LC62, and LC67) and their isogenic differentiated derivatives (5d-RAI and CM) (Supplemental File 3). Analysis of lineage-specific markers confirmed the identity of the three different cellular states (Fig. 2A). Reassuringly, the pluripotency genes *LIN28A*, *POU5F1*, *NANOG* and *SOX2* are expressed only in iPSCs [30]. Retinoic acid treatment induced markers of differentiated states such as *KRT19*, *TFAP2A*, *ID2*, *KLF3* and *DLX5* [31–35]. Conversely, in CMs we observed expression of muscle-specific genes such as myosins (e.g., *MYL7*, *MYH7*) and troponins (e.g., *TNNI3*, *TNNC1*) [36]. Notably, p53 induction with Nutlin does not affect expression of these markers of pluripotency and differentiation in this setting. Ingenuity Pathway Analysis (IPA) predicted multiple drivers of the transcriptome changes caused by the differentiation protocols (Fig. S2B, C, Supplemental File 4). iPSC differentiation by RA resulted in patterns indicative of downregulation of SOX3, SOX1, and alpha-catenin, along with activation of retinoic acid receptors (RARA, RARG), BMP2, and angiotensinogen (AGT). In contrast, iPSC differentiation into CMs showed signs of repression of the MYC transcriptional program, indicative of decreased cell proliferation, along with increased activity of MYOD1, MEF2C, and MYOCD, three well-characterized regulators of myogenic gene expression programs. In agreement with previous reports, we observed that differentiation of iPSCs blocks the apoptotic response caused by p53 activation as evidenced by the loss of cleaved caspase 3 and much-reduced levels of Annexin V/PI-positive cells (Fig. 2B, Fig. S2D, E) [37–39]. We then identified differentially expressed genes (DEGs) upon Nutlin-3 treatment in iPSCs, 5d-RAIs, and CMs. An overview of the DEGs in both the upregulated and downregulated categories revealed significant differences in the number of genes in each group for each cellular state (Fig. 2C–E, Supplemental File 3). For example, only 49 mRNAs were commonly induced by Nutlin-3 treatment in all three cellular states, with most mRNAs being induced in a cell type-specific manner (Fig. 2C). Moreover, a comparison of fold changes in expression of the DEGs identified in iPSCs versus the differentiated states displayed a substantial difference, with hundreds of genes being clearly impacted by Nutlin-3 treatment only in iPSCs (Fig. 2E).

Given that RNA-seq results could be impacted by changes in mRNA stability or degradation, we focused our analysis of well-characterized direct transcriptional p53 target genes [4, 40] (Fig. 1), which revealed high heterogeneity in their response to p53 activation in different cellular states (Fig. 2C–F). For example, *CDKN1A* was commonly induced in all three cellular states, but *TP53I3* was induced only in iPSCs and 5d-RAIs but not CMs. *PGPEP1*, a p53 target gene encoding an enzyme that cleaves amino terminal pyroglutamate [41], was induced in iPSCs and CMs but not in 5d-RAIs. *RAP2B* was induced in both differentiated cell lines (5d-RAIs and CMs) but not in iPSCs. *ATF3*, a transcription factor acting downstream of p53, was induced only in iPSCs [42]. *FBXW7*, which functions in phosphorylation-dependent ubiquitination processes [43] was induced only in 5d-RAIs. *LRP1*, a p53 target gene involved in endocytosis and signal transduction [44] was only induced in CMs (Fig. 2C–F). Ingenuity pathway analysis (IPA) of all the upregulated genes in all three isogenic cellular states revealed consistent activation of a p53 signature (Fig. 2G), predicting p53 as a top upstream regulator of the changes observed (Fig. 2H). However, this analysis also revealed high diversity in other pathways affected by Nutlin-3 treatment (Fig. 2G), potentially indicating that indirect responses downstream of p53 activation are also different across cellular states.

Altogether, these results demonstrate that cell differentiation strongly modifies the gene expression changes caused by p53 activation, which prompted us to investigate the potential underlying mechanisms.

### Cell differentiation reveals diverse regulatory classes within the p53 network

**Fig. 3:**
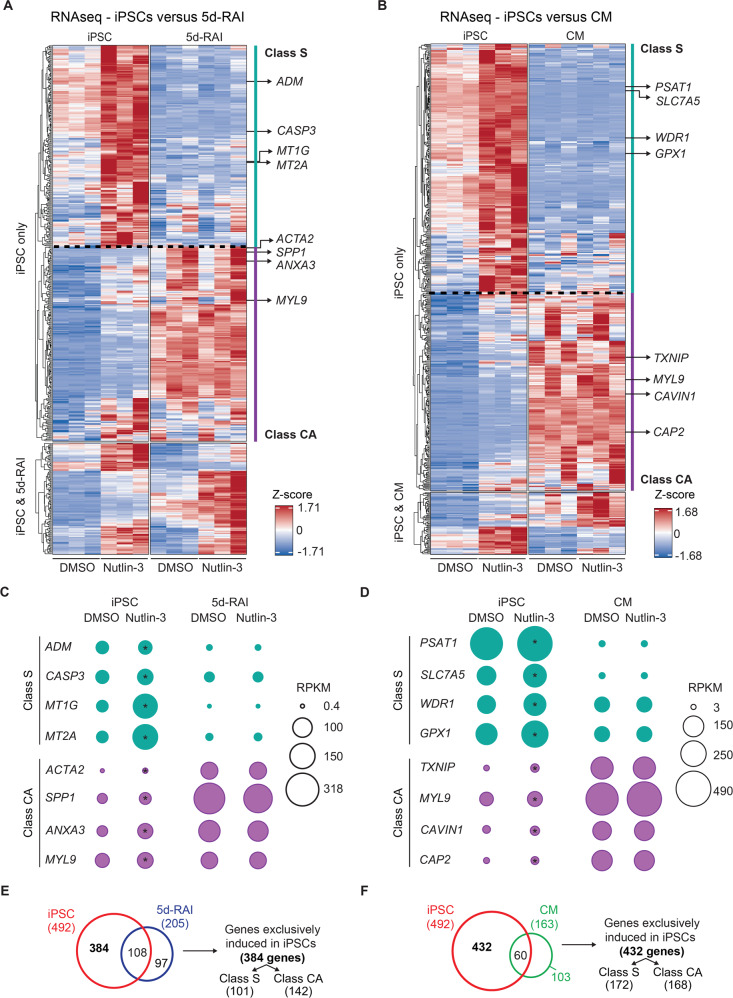
Cell differentiation reveals diverse regulatory classes within the p53 network. Heatmaps showing the relative expression levels (Z-scores of RPKMs) of genes induced by Nutlin-3 in iPSCs versus cells differentiated by retinoic acid treatment (5d-RAIs) (**A**) or cardiomyocytes (CMs) (**B**). The rows are split by the cell types in which the genes are induced. In both heatmaps, the “iPSC only” cluster indicates genes induced exclusively in iPSCs. The “iPSC & 5d-RAI” or “iPSC & CM” label indicates genes induced in iPSCs and each of the differentiated states, respectively. The dashed lines within the iPSC-only clusters indicated the major branching in the unsupervised clustering, leading to representation of Class S (silenced genes, green vertical line) and Class CA (constitutively active genes, purple vertical line), respectively. *ADM*, *CASP3*, *MT1G*, and *MT2A* are examples of genes induced in iPSCs but silenced in 5d-RAIs (**A**), and *PSTAT1*, *SLC7A5*, *WDR1*, and *GPX1* are examples of genes induced in iPSCs but silenced in CMs (**B**). *ACTA2*, *SPP1*, *ANXA3*, and *MYL9 are* examples of genes induced in iPSCs and upregulated at baseline in 5d-RAIs (**A**), and *TXNIP*, *MYL9*, *CAVIN1*, and *CAP2* are examples of genes induced in iPSCs and upregulated in CMs (**B**). **C**, **D** Bubble plots displaying examples of absolute expression values (in reads per kilobase per million, RPKM) of each of the gene classes in (**A** and **B**), respectively. Asterisks (*) denote statistically significant fold change by DESeq2 (*q* < 0.1) upon Nutlin-3 treatment. (**E**, **F**) Venn diagram displaying the identification scheme of the genes that are exclusively induced in iPSCs but not in 5d-RAIs (**E**) or CMs (**F**). All data shown from three different cell lines representing three biological replicates. See also Figure S3.

Having observed clear diversity in the gene expression changes upon p53 activation across different cellular states, we focused our analysis on genes upregulated upon Nutlin-3 treatment, as several lines of evidence indicate that gene repression downstream of p53 activation is mostly indirect [42, 45–48]. Unsupervised clustering analysis of all significantly induced genes upon Nutlin-3 treatment in iPSCs produced several interesting observations (Fig. 3A, B). First, the major two branches in the unsupervised clustering analysis revealed two distinct behaviors in terms of gene expression changes upon differentiation. One major cluster involves genes that are clearly silenced in the differentiated states, either 5d-RAI and/or CM. Examples include *ADM*, *CASP3*, *MT1G*, and *MT2A*, which are induced in iPSCs but silenced in 5d-RAI (Fig. 3A, C), and *PSTAT1*, *SLC7A5*, *WDR1*, and *GPX1*, which are induced in iPSCs but silenced in CMs (Fig. 3B, D). Second, many genes induced only in iPSCs were strongly upregulated at baseline in the differentiated states, suggesting a mechanism by which their expression is upregulated during cellular differentiation without operator-induced p53 activation. Examples of such ‘constitutively active’ genes include *ACTA2*, *SPP1*, *ANXA3*, and *MYL9* in 5d-RAIs (Fig. 3A, C), and *TXNIP*, *MYL9*, *CAVIN1*, and *CAP2* in CMs (Fig. 3B, D).

To further explore this phenomenon, we classified genes into distinct regulatory classes depending on their behavior in iPSCs versus the differentiated cellular states (Fig. 3E, F). These exercises identified 384 genes that were specifically induced in iPSCs but not in 5d-RAIs (Fig. 3E) and 432 genes that were specifically induced in iPSCs but not in CMs (Fig. 3F). Next, we classified the genes exclusively induced in iPSCs based on their behavior in the differentiated states into two major regulatory classes referred hereto as Class S (for Silenced) and Class CA (for Constitutively Active) (Fig. S3). We hypothesized that cellular differentiation results in silencing of a fraction of p53 target genes (Class S), likely via epigenetic silencing through the action of DNA methyltransferases (DNMTs) [49] or Polycomb Repressive Complexes (PRC1/2) [50–53]. For Class CA, we hypothesized that cell differentiation results in the activation of lineage-specific transcription factors (TFs) that regulate constitutive expression of a group of p53 target genes regardless of p53 activity. To investigate if this phenomenon could also affect gene sets regulated by other ubiquitous signaling pathways, we analyzed the behavior of a gene set induced upon hypoxia signaling [54] and a gene set induced downstream of interferon-gamma signaling [55]. In both cases, basal expression of many of these genes was affected by the differentiation protocols, with varying subsets being repressed and activated (Fig. S3B, C), indicating a general phenomenon that could impact other gene networks as well. From this point forth, we focused our efforts on investigating the underlying mechanisms modifying the p53 transcriptional program in the iPSC to 5d-RAI differentiation paradigm.

### Gene silencing blocks a large fraction of the p53 network upon cell differentiation

**Fig. 4:**
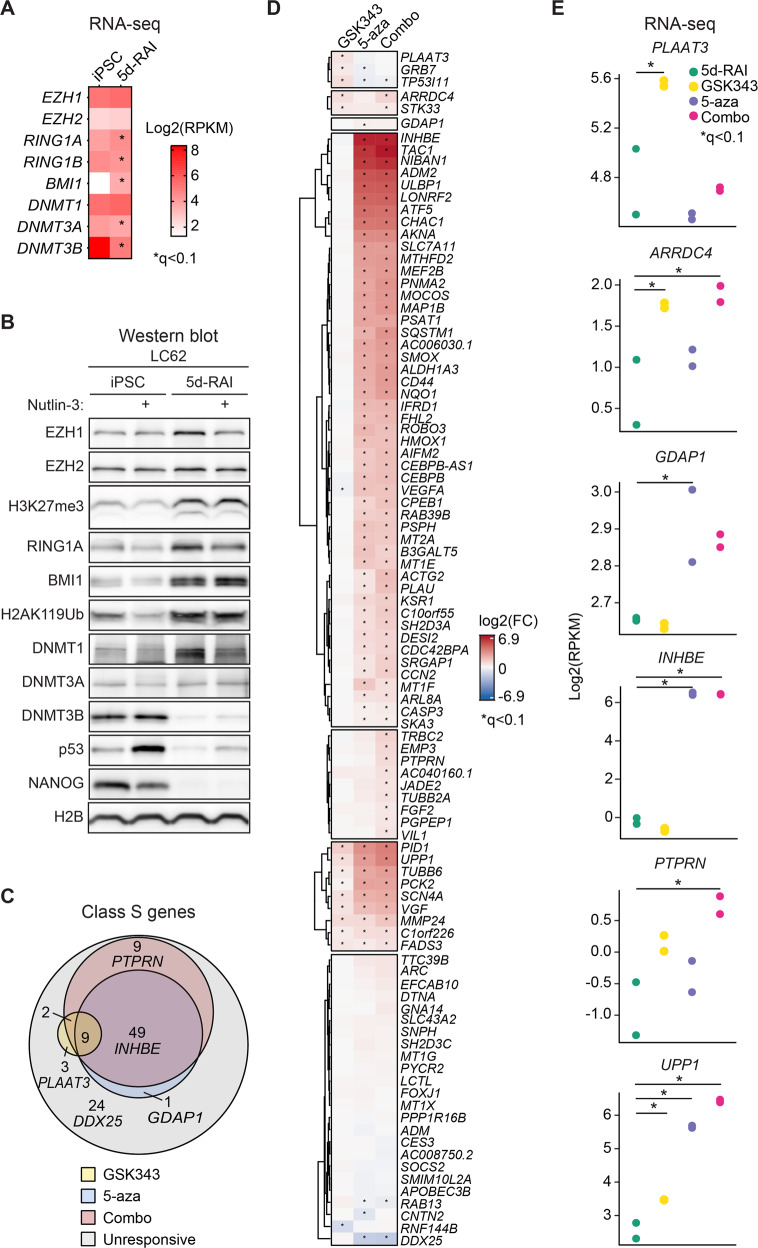
Gene silencing blocks a large fraction of the p53 transcriptional program upon cell differentiation. **A** Heatmap of absolute mRNA expression values (reads per kilobase per million, RPKM) in unstimulated iPSCs and their isogenic 5d-RAI derivatives candidate genes selected as potential regulators of Class S genes. Asterisks (*) denote statistically significant fold changes as defined by DESeq2 (*q* < 0.1). **B** Immunoblots of potential regulators of Class S genes in the cell line LC62, including subunits of the PRC2 complex (EZH1, EZH2), histone H3 lysine 27 tri-methylation (H3K27me3), subunits of the PRC1 complex (RING1A, BMI1), histone H2A lysine 119 ubiquitination (H2AK119Ub), DNA methyltransferases (DNMT1, DNMT3A, and DNMT3B), p53, the pluripotency marker NANOG, and the loading control histone H2B. Two biological replicates are shown in Figure S4. **C** Venn diagram representing subsets of Class S genes based on their rescued expression upon treatment with GSK343, 5-azacytidine (5-aza), or the combination of both drugs, and their classification into 4 major groups: derepressed by GSK343, derepressed by 5-aza, derepressed by the combination, and unresponsive. Placement into each category was defined by statistical significance as defined by DESeq2 (*q* < 0.1). RNA-seq was done from two biological replicates. **D** Heatmap representation of fold changes (FC) of Class S genes upon treatment with GSK343, 5-aza, or the combination of both drugs in the 5d-RAI differentiated state. RNA-seq was done from two biological replicates. Asterisks (*) denote statistically significant fold change (*q* < 0.1) based on DESeq2. (**E**) Plots displaying absolute gene expression (RPKM) of Class S genes belonging to the categories identified in panel C. RNA-seq was done from two biological replicates. Asterisks (*) denote statistically significant fold change (*q* < 0.1) based on DESeq2. See also Figure S4 and Supplemental File 5.

To test the involvement of epigenetic regulators in silencing of Class S genes, we first generated a candidate list of the potential regulators based on changes in their basal mRNA expression during the RA differentiation protocol. To this end, we mined the RNA-seq data for subunits of known gene silencing complexes whose expression would be modulated upon 5d-RAI differentiation, including subunits of the PRC1 and PRC2 complexes and DNA methyltransferases (DNMT1s) (Fig. 4A, B). This analysis revealed that the 5d-RAI differentiation protocol significantly increased mRNA expression of the PRC1 complex subunits RING1A and BMI1, with a trend toward increasing EZH1, EZH2, and DNMT1 expression. In contrast, the 5d-RAI protocol downregulated expression of RING1B, DNMT3A, and DNMT3B. Western blot analysis revealed increased levels of H2AK119Ub and H3K27me3, indicative of increased PRC1 and PRC2 activity, respectively (Fig. 4B, Fig. S4A, C). Western blots also confirmed increased levels of DNMT1 protein expression along with decreased levels of DNMT3B (Fig. 4B, Fig. S4A, B). Notably, we also observed a decrease in H2AK119Ub in Nutlin-treated iPSCs, albeit only for two of the isogenic pairs (Fig. 4B, Fig. S4A, B).

We next used pharmacological inhibitors of PRC2 and DNMT1 to assess the effect of blocking their enzymatic activities on the expression of Class S genes. PRC2 mediates H3K27me3 via its catalytic subunits EZH1 or EZH2, and their activity can be blocked with the specific small molecule inhibitor GSK343 [56, 57]. We exposed iPSCs to increasing concentrations of GSK343 over five days in parallel to RA treatment and examined expression levels of EZH1, EZH2 and H3K27me3 (Fig. S4C). While EZH1 and EZH2 protein expression remained mostly unchanged during the treatment period (except at the high concentration of 4 μM), H3K27me3 was strongly reduced to levels similar to those observed in undifferentiated cells, in a dose-dependent fashion (Fig. S4C). Importantly, GSK343 treatment did not restore NANOG expression nor clearly affect cellular morphology in lineages differentiated with RA (Fig. S4C, D), indicating that removal of the H3K27me3 mark is not sufficient to reverse differentiation in this setting. To block DNMT1 activity, we treated cells with the well-characterized small molecule inhibitor 5-azacitidine (5-aza) [58]. Then, we differentiated iPSCs with RA for 5 days and treated the cells with the single agents (GSK343 or 5-aza) or their combination (Combo) over the last two days of the differentiation protocol followed by polyA^+^ RNA-seq analysis to monitor the impact of treatments on gene expression changes among Class S genes. This analysis revealed the presence of several gene categories, including: (i) genes derepressed by single inhibition of PRC2, such as *PLAAT3* and *ARRDC4*, (ii) genes derepressed by inhibition of DNMT1, such as *GDAP1* and *INHBE*, (iii) those derepressed only upon dual inhibition of PRC2 and DNMT1, such as *PTPRN*, (iv) genes derepressed by inhibition of either PRC2 or DNMT1, such as *UPP1*, and (v) genes which remained repressed, or where even repressed further, upon treatment with the inhibitors, such as *DDX25*, indicative of other mechanisms involved in maintenance of their repressed state (Fig. 4C–E, Fig. S4E, Supplemental File 5).

Collectively, these results reveal the impact of epigenetic factors during differentiation-driven transcriptional silencing of a fraction of p53 transcriptional program.

### Lineage-specific transcription factors impact the p53 network

**Fig. 5:**
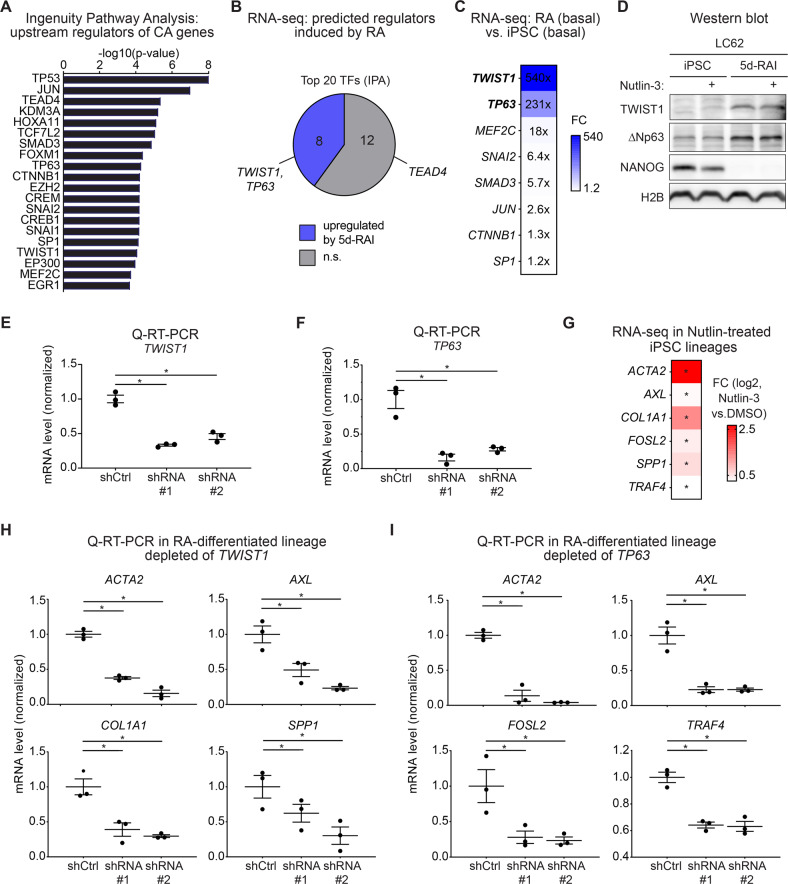
Expression of constitutively active p53 target genes is driven by lineage-specific transcription factors. **A** Top 20 transcription factors predicted as upstream regulators of Class CA genes by Ingenuity Pathway Analysis (IPA) ranked by decreasing significance. Only transcription factors upregulated in 5d-RAI with z-activation score > 1 and *q* < 0.1 are displayed. p values are calculated by IPA using a hypergeometric test. **B** Ven diagram showing the fold changes (FC) of the top 20 predicted upstream regulators from **A**, 8 of which were significantly upregulated at the basal level in 5d-RAIs relative to iPSCs. Significance was defined by DESeq2 (*q* < 0.1) from data generated from three cell lines representing three biological replicates. n.s., not significant. **C** Heatmap displaying the fold change in basal expression of indicated mRNAs in 5d-RAI relative to iPSCs, showing *TWIST1* and *TP63* as the top predicted transcription factors with the largest fold change. **D** Immunoblots of cell line LC62 showing increased TWIST1 and ∆Np63 protein expression upon cell differentiation. Two biological replicates are shown in Figure S5. (**E**, **F**) Q-RT-PCR analysis showing decreased mRNA expression of *TWIST1* (E) and *∆Np63* (F) upon knockdown with two independent shRNAs relative to the non-targeting control (shCtrl) in 5d-RAIs. An asterisk denotes a statistically significant difference at p < 0.05 calculated using *t*-test. Data are presented as S.E.M. (*n* = 3), normalized to shCtrl. (**G**) Heatmap displaying induction of selected p53 target genes from the Class CA predicted to be coregulated by TWIST1 or ∆Np63 as measured by RNA-seq. Significance was defined by DESeq2 (*q* < 0.1) from data generated from three cell lines representing three biological replicates. Plots demonstrating the impact of knocking down of *TWIST1* (**H**) and *∆Np63* (**I**) on the group of genes predicted to be activated by these transcription factors with two independent shRNAs in 5d-RAI cells as measured by Q-RT-PCR. Data is from three biological replicates. An asterisk denotes a statistically significant difference at p < 0.05 calculated using *t*-test. Data are normalized to shCtrl values and presented as S.E.M. (*n* = 3). See also Figure S5.

To identify potential lineage-specific TFs driving expression of the Class CA genes, we used IPA to predict upstream regulators of this gene class. Expectedly, *TP53* was identified as the top predicted upstream regulator, consistent with the fact that these genes were induced by Nutlin-3 in iPSCs (Fig. 5A). Other predicted upstream regulators include AP-1 family members (e.g., *JUN*), the regulator of the HIPP pathway *TEAD4*; and several chromatin-modifying enzymes (e.g., *KDM3A*, *EP300*), among others (Fig. 5A). To prioritize this candidate factor list, we tested which of these genes were strongly upregulated at the mRNA level in 5d-RAI while also exhibiting a positive activation score in IPA (Fig. 5A, B). Of the top twenty predicted upstream regulators, 8 of them were significantly upregulated in 5d-RAIs relative to iPSCs (Fig. 5B), with *TWIST1* and *TP63* showing the strongest upregulation (Fig. 5C, Fig. S5A–C). Interestingly, using available chromatin occupancy data [59], we found increased frequency of both p63 and TWIST1 chromatin binding sites near the transcription start sites of CA genes relative to S genes or all genes induced by Nutlin in iPSCs only (Fig. S5D). Isoform analysis demonstrated that ∆*N*p*63* was the main mRNA upregulated from the *TP63* locus upon differentiation (Fig. S5A, E). Upregulation of *TWIST1* and *∆N*p*63* was also observed at the protein level (Fig. 5D, Fig. S5C). To examine the involvement of TWIST1 or ∆Np63 in expression of the Class CA genes, we generated iPSC lines constitutively expressing shRNAs targeting either factor and monitored the impact of their knockdown on genes predicted to be activated by these transcription factors (Fig. 5E–G, Fig. S5B). Indeed, expression of numerous Class CA genes was diminished in cells depleted of TWIST1 and/or ∆Np63 (Fig. 5H, I). For example, *ACTA2*, *AXL*, *COL1A1, and SPP1*, all of which were significantly induced by Nutlin-3 in iPSCs and significantly upregulated by the differentiation protocol (Fig. 5G), were statistically downregulated upon knockdown of *TWIST1* with two independent shRNAs (Fig. 5H). Likewise, *ACTA2*, *AXL, FOSL2*, and *TRAF4* were significantly downregulated upon *∆N*p*63* knockdown with two independent shRNAs (Fig. 5I). Notably, depletion of TWIST1 or ∆Np63 did not restore expression of NANOG, indicating that their induction is not required to sustain the differentiated state (Fig. S5F, G).

Altogether, these results demonstrate that cell differentiation can modify the fraction of the transcriptome that is responsive to p53 activation via constitutive transactivation by other transcription factors.

## Discussion

Despite the undisputed importance of p53 in cancer biology, the mechanisms by which different cellular states impact the p53 network remain to be fully elucidated. For instance, it is unclear how the p53 transcriptional program is modulated in different untransformed cellular lineages within the human body. Genome-wide analysis suggests high conservation of p53 chromatin binding sites, but transcriptome analysis shows vastly non-overlapping sets of p53 target genes across cell types [40]. Here, we report that cellular differentiation restrains a large fraction of the p53 program in a process involving epigenetic silencing, as demonstrated by rescued expression by direct inhibition of PRC2 and DNMT1 enzymatic activities. However, our results indicate the presence of additional repressive mechanisms that could affect the p53 network, such as histone deacetylases (HDACs), nucleosome sliding factors (e.g., SWI/SNF complexes), repressive non-coding RNAs [60], or even perhaps the lack of p53 transcriptional co-factors required for coactivation of some target genes in differentiated cells [61]. Nevertheless, our results point to DNA methylation as the most prevalent mechanism restraining the p53 network upon differentiation. Noteworthy, p53 itself could modulate the action of various epigenetic silencing complexes, which in turn could affect its own transcriptional network. For example, previous reports have documented induction of EZH1 expression as well as indirect downregulation of EZH2, DNMT1, and DNMT3B upon p53 activation [45–47]. We also found that cellular differentiation induces a sizable fraction of the p53 network in a process involving lineage-specific transcription factors. We showed that TWIST1 and *∆*Np63 co-regulate a subset of p53 target genes, contributing to their induction upon differentiation, suggesting that these two transcription factors commonly bind to a set of p53 target gene promoters and/or enhancers. For p63, a member of the p53 family of DNA-binding proteins, this notion is clearly supported by previous work [62–64]. Noteworthy, gene silencing and constitutive transactivation upon cell differentiation are likely to impact the action of other master transcriptional regulators expressed in both stem cells and differentiated lineages, as illustrated for hypoxia-inducible genes and genes responsive to interferon gamma (Fig. S3B, C).

We hypothesize that understanding how cell differentiation impacts the p53 transcriptional network would improve the design of p53-based cancer therapies. Most tumors display great cellular heterogeneity, with the existence of both cancer stem cells (CSCs) and various differentiated lineages [65]. The stem-like properties of CSCs may promote higher oncogenic plasticity, metastasis, and drug resistance [66–68]. We showed here that cell differentiation modulates and restrains the p53 program, thus it is possible that the undifferentiated state of CSCs would open a fraction of the p53 transcriptional program with potential to enhance its tumor suppressive function. Therefore, a greater understanding of the impact of cell differentiation on the p53 transcriptional program may inform the use of p53-activating agents in combination with agents that modulate cell differentiation [69, 70].

In conclusion, this study provides new insights on the complexity of the transcriptome changes induced by p53 activation in non-transformed cells and provides deeper understanding of the impact of cell differentiation on the p53 network in a cell type-specific context, while also increasing our general knowledge of gene expression control mechanisms.

## Methods and Materials

### Sample sizes, sample collection, replicates, statistical methods, and blinding

For gene expression and genetic co-dependency analysis in Fig. 1, samples sizes were defined by the datasets available from the GTEx and DepMap projects (see details below). For all iPSC work, including RNA-seq, Western Blots, apoptotic index assays, shRNA knockdown, and Q-RT-PCRs, results were obtained from three different cell lines representing three different biological replicates, or from three different biological replicates from the same cell line, as appropriate for each experiment and as indicated in the respective figure legends. All statistical methods and number of replicates are indicated in each figure legend and described in detail below for each type of experiment. The identity of cell cultures or treatment conditions were not blinded to operators, except for generation of RNA-seq data, where the sequencing center was blinded to experimental conditions.

### Bioinformatic analyses of GTEx and DepMap datasets

Genome-wide RNA-seq data from non-diseased (normal) tissues were obtained from the Genotype-Tissue Expression (GTEx) project (Version 8, https://gtexportal.org/home/datasets, accessed on 4/22/22). Tissues with <10 representative samples were excluded, resulting in 29 lineages for our analysis. TPM values for 103 core p53 target genes [4] were log2 adjusted (log2(TPM + 1)). “Relative expression” of each individual gene across lineages was determined by normalizing the median lineage-specific expression to that of the tissue with the highest expression of that gene. RNA-seq data (log2(TPM + 1) values) from 1393 cancer cell lines, encompassing 38 distinct lineages, were obtained from The Cancer Dependency Map (DepMap) project (Version 22Q1, https://depmap.org/portal/download/all/, accessed on 3/30/22). Engineered cell lines, lines without lineage information, and lineages with less than 10 representative samples were excluded from the analysis leading to 1374 cell lines encompassing 26 lineages. Relative expression of the same 103 p53 target genes was determined across these 26 cancer lineages. Relative expression matrices of both normal tissues and cancer cell lines for all 103 genes in addition to 20 select genes of interest were visualized by unsupervised hierarchical clustering. Non-pairwise Wilcoxon rank sum testing was used to compare expression of genes across different lineages, followed by Benjamini-Hochberg correction to control for false-discovery (q-values). Computations and visualizations were performed in R (R v4.0.3; R Studio 1.4.1103) using a custom script and the tidyverse (v1.3.0), ComplexHeatmap (v2.6.2) and ggplot2 (v3.3.3) packages.

Genome-wide gene effect scores derived from CRISPR knockout screens and inferenced by Chronos for 17386 genes across 1070 cell lines were obtained from the DepMap project (Version 22Q1, https://depmap.org/portal/download/all/, accessed on 3/30/22). As per DepMap guidance, gene effect scores < −0.5 were considered essential, whereas the median gene effect score of all common essential genes is −1.0. Genetic co-dependency between all genes in the dataset was assessed by computing pairwise Spearman correlations and p-values of gene effect scores, followed by Benjamini-Hochberg correction to control for false-discovery (q-values). The gene effect score distributions of 103 core p53 target genes were ranked by their median gene effect scores. Computations and visualizations were performed in R (R v4.0.3; R Studio 1.4.1103) using a custom script and the tidyverse (v1.3.0), ComplexHeatmap (v2.6.2) and ggplot2 (v3.3.3) and rstatix (v0.7.0) packages.

### Human iPSC Generation

Human urine samples were obtained through the Crnic Institute Human Trisome Project Biobank (NCT02864108). Sample collection was approved by the Colorado Multiple Institutional Review Board (COMIRB protocol #15-2170) and written consent was obtained from all participants or their legal guardians. Human iPSCs (*n* = 3) were generated from amplified kidney epithelial cells at the Stem Cell Biobank and Disease Modeling Core Facility of the Gates Center for Regenerative Medicine using a RNA-based reprogramming protocol as previously published [71]. Briefly, kidney epithelia cells isolated from urine samples were reprogrammed with a cocktail of six human transcription factors delivered as modified mRNAs (mod-mRNAs) known as “5fM3O mod-mRNAs”, consisting of M3O (OCT4 fused with the MyoD transactivation domain) [72], SOX2, KLF4, cMYC, NANOG, and LIN28A, along with reprogramming mimics miRNAs 302a/b/c/d and 367. The iPSC lines are labeled as LC62, LC42, and LC67. All iPSC lines showed normal karyotype as determined by G-banding analysis performed by WiCell.

### Cell culture

Human iPSC lines were cultured on Matrigel (Corning, 54277, NY, USA) coated plates, and were maintained in mTeSR1 media (Stem Cell Technology, 85850, Canada) supplemented with 1% Antibiotic-Antimycotic mix Solution (Caisson Labs, ABL02-6, UT, USA) at 37 °C, 5% O_2_ and 5% CO_2_. iPSCs received fresh media daily and were passaged every 4-5 days. HEK293FT packing cells (ATCC, CRL-3249) were maintained in DMEM complete media (Fisher Scientific, 11-995-073, NY, USA) supplemented with 10% FBS (Peak Serum, PS-FB3, CO, USA) and 1% Antibiotic-Antimycotic mix Solution at 37°C and 5% CO_2_. All the cell lines were tested biweekly for mycoplasma contamination by PCR.

### Generation of isogenic differentiated cell lines and Nutlin treatment

iPSC lines were induced into monolayer non-specific lineage in the presence of 10 µM

all-*trans-*Retinoic Acid (Sigma-Aldrich, R2625, MO, USA) in mTeSR1 media on Matrigel coated plates for 5 days (5d-RAIs), with daily fresh media change. At day 5 of the differentiation protocol cells were treated with 2.5 µM Nutlin-3R (Cayman Chemical Company, 10004372, MI, USA) or DMSO for 12 h in mTeSR1 media supplemented with 10 µM RA to activate p53. Cardiomyocytes (CM) were generated using published protocols [73]. Briefly, iPSCs were induced into monolayer CMs using DMEF media supplemented with B27, GSKb inhibitors, and WNT inhibitors for 45 days at 37°C and 5% CO_2_. The CMs were selected from none-cardiomyocytes cells using glucose-free DMEM (no glucose, no pyruvate; Invitrogen, Canada) supplemented with 4 mM lactate. To avoid differences imposed by culture conditions (oxygen concentration and media), CMs were incubated in mTeSR1 media at 37°C, 5% CO_2_, and 5% O_2_ for 24 h prior to Nutlin-3 treatment. All experiments were performed with cells within 8 passages.

### shRNA-mediated knockdown

iPSCs were stably transduced with two independent shRNAs targeting *TWIST1* (sh-TWIST1) or *TP63* (shTP63) mRNAs using a lentiviral-based delivery system. shRNAs were expressed from the pLKO.1 plasmid (TRC library, Sigma-Aldrich, St. Louis, MO) obtained from University of Colorado Cancer Center Functional Genomics Shared Resource (University of Colorado Anschutz Medical Campus, CO, USA). For lentivirus generation, the HEK293FT cell line was used for lentiviral packaging. 24 h prior to transfection, HEK293FT cells were passaged from 70% confluent 150-mm petri dishes in 1 to 8 ratios in DMEM complete media using 10 cm petri dishes to reach confluency of 70-75% the next day. Transfection was performed using 3 µg of packaging plasmids consisting of 2 µg of psPAX2 (Addgene, 12260) and 1 µg of pMD2.G (Addgene, 12259), and 2 µg of shRNA vector, transfected into the host cells with 12 μg of polyethyleneimine (PEI, Polyscience, 23966) in Opti-MEM Reduced Serum Media (Thermo Fisher Scientific, 31985062, NY, USA). 14-16 h after transfection cells were fed with fresh DMEM complete media and incubated for 48 h to produce virus particles. Media was collected and filtered with a 0.45-micron cellulose acetate filter. Cells were transduced in the presence of 8 µg/ml polybrene (Sigma-Aldrich, H9268, MO, USA) for 16–18 h at 37 °C, 5% O_2_, and 5% CO_2_. Two days after transduction, colonies were selected in the presence of 1.5–2 µg/ml puromycin (Research Product international, P33020, IL, USA). *SHC002* puromycin resistance shRNA expressed in pLKO.1 plasmid with non-mammalian target site from TRC library (TRC library, Sigma-Aldrich, St. Louis, MO) was used as a control, noted as shCtrl. The efficiency of knockdown was confirmed by Q-RT-PCR against the target mRNA and Western blot. The shRNA sequences were used are presented in Supplemental Table 1.

### Drugs and drug treatments

Nutlin-3R (Cayman Chemical Company, 10004372, MI, USA) and GSK343 (Med Chem Express, HY-13500) were dissolved in DMSO at 10 mM concentration, aliquoted, and stored at -80°C. 5-azacytidine (Sigma Aldrich, A2385) was dissolved in water at concentration of 10 mM, and aliquots stored in –80°C for single use. All-*trans* retinoic acid (Sigma, R2625) was dissolved in DMSO at 0.133 M and aliquots were stored in –80°C. Puromycin (Sigma, P8833) was dissolved in water at 10 mg/mL, aliquoted, and stored at -20°C.

All the Nutlin-3 treatments were performed at final concentration of 2.5 µM for 12 h at 37 °C, 5% O_2_ and 5% CO_2_. 4 µM GSK343 and 5 µM 5-azacytidine individual treatments or their combination were applied to the 5d-RAI cells during the last two days of the 5-day all-*trans* retinoic acid induced differentiation protocol.

### Q-RT-PCR

Total RNA was extracted using TRIzol Reagent (Thermo Fisher Scientific, 15596018, CA, USA) following manufacturer’s instructions. RNA concentration and quality were verified using Take3 microplate reader (BioTek Synergy2, Gen5 1.11, Biotech Agilent, VT, USA) at absorbance of 260 nm. 250–300 µg of total RNA were used for reverse transcription cDNA reaction using High-Capacity cDNA Reverse Transcription Kit (Life Technologies, 4368813, Vilnius, Lithuania) as per manufacturer’s instructions. Quantitative PCR was performed with reference to standard curve prepared from the pooled cDNA samples using SYBER Select Master Mix CFX (Thermo Fisher Scientific, 4472954) on a Viia7 Real-Time PCR system (Thermo Fisher Scientific, Vilnius, Lithuania). 18S rRNA was used as an internal control for normalization. Three independent biological replicates were conducted for each of the iPSC and its isogenic lineages. The primer sequences were presented in the Supplementary Table 2.

### Histone acid extraction

Histone acid extraction was performed as described before [74]. Briefly, approximately 5 × 10^6^ cells/ml were collected by scrapping off the cells into ice-cold PBS and spun at 300 x *g* for 4 min. Cell pellets were washed once with 5 ml of cold PBS and collected at 300 x *g* for 4 min. Cell pellets were resuspended in 1 ml of cold hypotonic lysis buffer (10 mM Tris-HCl pH 8.0. 1 mM KCl, 1.5 mM MgCl_2_, 1 mM DTT), protease inhibitors (Roche, 1697-498) and phosphatase inhibitors (PhosSTOP, Roche, 94906837001, Mannheim Germany) using 1.5 ml microcentrifuge tubes and incubated at 4 °C for 30 min on the rotator to promote hypotonic swelling and cell lysis by mechanical shearing during rotation. Cell nuclei were collected at 10,000 x *g*, 10 min at 4 °C. To extract histones, nuclei were resuspended in 400 µl of 0.4 N sulfuric acid (0.4 N is equivalent to 0.2 molar) and incubated on rotor for 1 h at 4 °C. Samples were centrifugated to remove nuclear debris at 16,000 x *g* for 10 min a 4 °C (i.e., supernatant contained the histone proteins). After transferring the supernatant to the new 1.5 ml microcentrifuge tubes, histones were precipitated in the presence of equal volume of 50% (w/v) TCA (25% final concentration) and incubated at 4 °C for 1 h to remove acid residues from the histone pellets, then 1 ml of ice-cold acetone was added to the histone pellets and incubated on ice for 10 min. Histone pellets were collected at 16,000 x *g* for 10 min at 4 °C and air-dried at room temperature for 20 min. Histone pellets were dissolved in 80–100 µl of water and its concentration was quantified using BCA Protein Assay Kit (Pierce, Thermo Fisher Scientific, 23225, IL, USA) following the manufacturer’s instructions. 1.5 µg of histones per sample were resolved by 12% polyacrylamide gel electrophoresis and transferred onto 0.45 µm PVDF membrane (Thermo Fisher Scientific, 88518, IL, USA) and blocked with 5% BSA dissolved in TBS-T buffer (10 mM Tris pH 8.0, 150 mM NaCl, 0.1% v/v Tween-20). The histone subunit of interest was labeled with primary anti-histone antibodies in 5% BSA dissolved in TBS-T overnight at 4 °C. Next day the membrane was washed with TBS-T and membranes were incubated for 1 h with HRP-conjugated secondary antibodies on a rocker at room temperature and washed with TBS-T buffer for 5 min three times at room temperature. Detection was done with SuperSignal West Pico Plus Chemiluminescence Substrate (Pierce, Thermo Fisher Scientific, 34577, IL, USA) and digital images were acquired using ImageQuant LAS 4000 (GE Healthcare Life Sciences, Uppsala, Sweden).

### Western blots

Cell pellets were collected at 300 x *g* for 3 min at 4 °C. Pellets were washed twice with cold PBS and each time cell pellets were collected at 300 x *g* for 3 min at 4 °C. Cells were lysed on ice with cold RIPA buffer containing (150 mM NaCl, 1% v/v Igepal C630 NP-40, 0.5% w/v sodium deoxycholate, 0.1% w/v SDS,1 mM EDTA pH 8.0, 50 mM Tris HCl pH 8.0, and protease/phosphatase inhibitors) sonicated at 2.5 W for 10 s, heat-denatured in SDS-loading buffer (50 mM Tris HCl pH 6.8, 2% w/v SDS, 10% v/v glycerol, 1% 2-mercaptoethanol, 0.01% w/v bromophenol blue) for 3 min at 95 °C, and the protein concentration was quantified using a BCA Protein Assay Kit. 10-20 µg of total protein per sample were resolved by SDS-PAGE, transferred onto 0.45 µm PVDF membranes by electrophoresis, and blocked with 5% w/v BSA powder in TBST buffer (10 mM Tris pH 8.0, 150 mM NaCl, 0.1% v/v Tween 20). Proteins of interest were labeled overnight at 4 °C with primary antibodies in 5% BSA/TBST. Membranes were washed three times with TBST for 10 min, incubated with HRP-conjugated secondary antibodies for 1 h, and again washed three times in TBST. SuperSignal West Pico Plus Chemiluminescence Substrate was used for detection and digital images were captured using an ImageQuant LAS 4000. Antibodies used in this study were presented in Supplemental Table 3. The original uncropped western blots are provided in Supplemental File 6.

### RNA-seq

For the RNAseq experiment presented in Fig. 2, total RNA was extracted from three hiPSC lines (LC42, LC62, and LC67), and their isogenic differentiated derivative (5d-RAIs, CMs) treated for 12 h with either vehicle (DMSO) or 2.5 µM Nutlin-3R using TRIzol Reagent following manufacturer’s instructions. For RNA-seq experiment presented in Fig. 4, iPSCs very differentiated into 5d-RAI and treated with 4 µM GSK343, 5 µM 5-azacytidine, or their combination treatments during the last two days of the differentiation protocol followed by treatment with either vehicle (DMSO). Total RNA extracted using TRIzol Reagent following manufacturer’s instructions. RNA quality was assessed by Agilent Bioanalyzer 2100 (Agilent Technologies, G2939A, Germany) using RNA 6000 Pico chips (Agilent Technologies, 5067-1513, Germany), and concentration was measured by Qubit 2.0 Fluorometer (Life Technologies, Q32866, Singapore). We selected samples with RNA integrity number (RIN) equal or greater than 9.

### RNA-seq data analysis

For both of RNA-seq experiments, data from all the samples yield ~31-37 × 10^6^ final mapped reads per sample. Reads were demultiplexed and converted to fastq format using bcl2fastq (bcl2fastq v2.20.0.422). Data quality was assessed using FASTQC (v0.11.5) (https://www.bioinformatics.babraham.ac.uk/projects/fastqc/) and FastQ Screen (v0.11.0, https://www.bioinformatics.babraham.ac.uk/projects/fastq_screen/). Trimming and filtering of low-quality reads was performed using bbduk from BBTools (v37.99) [75] and fastq-mcf from ea-utils (v1.05, https://expressionanalysis.github.io/ea-utils/). Alignment to the human reference genome (GRCh38) was carried out using HISAT2 (v2.1.0) [76] in paired, spliced-alignment mode with a GRCh38 index and a Gencode v33 annotation GTF, and alignments were sorted and filtered for mapping quality (MAPQ > 10) using Samtools (v1.5) [77]. Gene-level count data were quantified using HTSeq-count (v0.6.1) [78] with the following options (–stranded=reverse –minaqual=10 –type=exon -mode=intersectionnonempty) using a Gencode v33 GTF annotation file. Differential gene expression was evaluated using DESeq2 (version 1.28.1) [79] in R (version 4.0.1), with Benjamini-Hochberg correction and *q* < 0.1 (FDR < 10%) as the threshold for differentially expressed genes.

### Flow cytometry

Treated cells were harvested by trypsinization, pelleted by centrifugation (200 *g* for 5 min at room temperature), and resuspended in annexin-V binding buffer (10 mM HEPES pH 7.4, 140 mM NaCl, 2.5 mM CaCl_2_). Approximately 2*10^5^ cells were labeled with Annexin V-FITC (Invitrogen) and propidium iodide (10 μg/ml, Millipore-Sigma) for 15 min in the dark before flow cytometric analysis (Accuri C6, Becton Dickinson).

### Statistical analysis

For Q-RT-PCR data, plot generation and statistical analysis was carried out using GraphPad Prism 9.3.1 (GraphPad Software, San Diego, CA, USA). Two-tailed unpaired Student *t*-test was used to compare between treatment groups. Error bars present the standard error of mean (SEM). Three independent biological replicates (*n* = 3) were performed for each of condition. Statistical significance of *p < 0.05 was used for all analyses.

## Supplementary information

Supplemental Material

Supplemental Table 1

Supplemental Table 2

Supplemental Table 3

Supplemental File 1

Supplemental File 2

Supplemental File 3

Supplemental File 4

Supplemental File 5

Original Data Files

Reproducibility checklist

## Data Availability

All relevant data that supports this work are available in the Supplemental Files. The transcriptome datasets generated during this study are available at Gene Expression Omnibus (GEO) database under accession number GSE207377.

## References

[1] Aubrey BJ, Strasser A, Kelly GL (2016). Tumor-suppressor functions of the TP53 pathway. Cold Spring Harb Perspect Med.

[2] Menendez D, Nguyen TA, Freudenberg JM, Mathew VJ, Anderson CW, Jothi R (2013). Diverse stresses dramatically alter genome-wide p53 binding and transactivation landscape in human cancer cells. Nucleic Acids Res.

[3] Vogelstein B, Lane D, Levine AJ (2000). Surfing the p53 network. Nature.

[4] Andrysik Z, Galbraith MD, Guarnieri AL, Zaccara S, Sullivan KD, Pandey A (2017). Identification of a core TP53 transcriptional program with highly distributed tumor suppressive activity. Genome Res.

[5] Levine AJ (1997). p53, the cellular gatekeeper for growth and division. Cell.

[6] Rufini A, Tucci P, Celardo I, Melino G (2013). Senescence and aging: the critical roles of p53. Oncogene.

[7] Kubbutat MH, Jones SN, Vousden KH (1997). Regulation of p53 stability by Mdm2. Nature.

[8] Li Q, Lozano G (2013). Molecular pathways: targeting Mdm2 and Mdm4 in cancer therapy. Clin Cancer Res: Off J Am Assoc Cancer Res.

[9] Vousden KH, Prives C (2009). Blinded by the light: the growing complexity of p53. Cell.

[10] Meek DW, Anderson CW (2009). Posttranslational modification of p53: cooperative integrators of function. Cold Spring Harb Perspect Biol.

[11] Beckerman R, Prives C (2010). Transcriptional regulation by p53. Cold Spring Harb Perspect Biol.

[12] Friedmann-Morvinski D, Verma IM (2014). Dedifferentiation and reprogramming: origins of cancer stem cells. EMBO Rep.

[13] Hollstein M, Sidransky D, Vogelstein B, Harris CC (1991). p53 mutations in human cancers. Science.

[14] Soussi T, Kato S, Levy PP, Ishioka C (2005). Reassessment of the TP53 mutation database in human disease by data mining with a library of TP53 missense mutations. Hum Mutat.

[15] Kandoth C, McLellan MD, Vandin F, Ye K, Niu B, Lu C (2013). Mutational landscape and significance across 12 major cancer types. Nature.

[16] Toledo F, Wahl GM (2006). Regulating the p53 pathway: in vitro hypotheses, in vivo veritas. Nat Rev Cancer.

[17] Dobbelstein M, Levine AJ (2020). Mdm2: Open questions. Cancer Sci.

[18] Vassilev LT, Vu BT, Graves B, Carvajal D, Podlaski F, Filipovic Z (2004). In vivo activation of the p53 pathway by small-molecule antagonists of MDM2. Science.

[19] Hafner A, Kublo L, Tsabar M, Lahav G, Stewart-Ornstein J (2020). Identification of universal and cell-type specific p53 DNA binding. BMC Mol Cell Biol.

[20] Stewart-Ornstein J, Iwamoto Y, Miller MA, Prytyskach MA, Ferretti S, Holzer P (2021). p53 dynamics vary between tissues and are linked with radiation sensitivity. Nat Commun.

[21] Fei P, Bernhard EJ, El-Deiry WS (2002). Tissue-specific induction of p53 targets in vivo. Cancer Res.

[22] Ringshausen I, O’Shea CC, Finch AJ, Swigart LB, Evan GI (2006). Mdm2 is critically and continuously required to suppress lethal p53 activity in vivo. Cancer cell.

[23] Cheng JC, Chang HM, Leung PC (2011). Wild-type p53 attenuates cancer cell motility by inducing growth differentiation factor-15 expression. Endocrinology.

[24] Johansson BB, Fjeld K, El Jellas K, Gravdal A, Dalva M, Tjora E (2018). The role of the carboxyl ester lipase (CEL) gene in pancreatic disease. Pancreatology.

[25] Fattahi F, Saeednejad Zanjani L, Habibi Shams Z, Kiani J, Mehrazma M, Najafi M (2021). High expression of DNA damage-inducible transcript 4 (DDIT4) is associated with advanced pathological features in the patients with colorectal cancer. Sci Rep.

[26] Kawase T, Ohki R, Shibata T, Tsutsumi S, Kamimura N, Inazawa J (2009). PH domain-only protein PHLDA3 is a p53-regulated repressor of Akt. Cell.

[27] Di J, Huang H, Qu D, Tang J, Cao W, Lu Z (2015). Rap2B promotes proliferation, migration, and invasion of human breast cancer through calcium-related ERK1/2 signaling pathway. Sci Rep.

[28] Allen MA, Andrysik Z, Dengler VL, Mellert HS, Guarnieri A, Freeman JA (2014). Global analysis of p53-regulated transcription identifies its direct targets and unexpected regulatory mechanisms. Elife.

[29] Tovar C, Rosinski J, Filipovic Z, Higgins B, Kolinsky K, Hilton H (2006). Small-molecule MDM2 antagonists reveal aberrant p53 signaling in cancer: implications for therapy. Proc Natl Acad Sci USA.

[30] Kashyap V, Rezende NC, Scotland KB, Shaffer SM, Persson JL, Gudas LJ (2009). Regulation of stem cell pluripotency and differentiation involves a mutual regulatory circuit of the NANOG, OCT4, and SOX2 pluripotency transcription factors with polycomb repressive complexes and stem cell microRNAs. Stem Cells Dev.

[31] Torma H (2011). Regulation of keratin expression by retinoids. Dermato Endocrinol.

[32] Krendl C, Shaposhnikov D, Rishko V, Ori C, Ziegenhain C, Sass S (2017). GATA2/3-TFAP2A/C transcription factor network couples human pluripotent stem cell differentiation to trophectoderm with repression of pluripotency. Proc Natl Acad Sci USA.

[33] Zhang J, Gao Y, Yu M, Wu H, Ai Z, Wu Y (2015). Retinoic acid induces embryonic stem cell differentiation by altering both encoding RNA and microRNA expression. PLoS One.

[34] Bruce SJ, Gardiner BB, Burke LJ, Gongora MM, Grimmond SM, Perkins AC (2007). Dynamic transcription programs during ES cell differentiation towards mesoderm in serum versus serum-freeBMP4 culture. BMC Genom.

[35] Yang L, Zhang H, Hu G, Wang H, Abate-Shen C, Shen MM (1998). An early phase of embryonic Dlx5 expression defines the rostral boundary of the neural plate. J Neurosci: Off J Soc Neurosci.

[36] Xu XQ, Soo SY, Sun W, Zweigerdt R (2009). Global expression profile of highly enriched cardiomyocytes derived from human embryonic stem cells. Stem Cells.

[37] Liu JC, Guan X, Ryan JA, Rivera AG, Mock C, Agrawal V (2013). High mitochondrial priming sensitizes hESCs to DNA-damage-induced apoptosis. Cell Stem Cell.

[38] Qin H, Yu T, Qing T, Liu Y, Zhao Y, Cai J (2007). Regulation of apoptosis and differentiation by p53 in human embryonic stem cells. J Biol Chem.

[39] Setoguchi K, TeSlaa T, Koehler CM, Teitell MA (2016). P53 regulates rapid apoptosis in human pluripotent stem cells. J Mol Biol.

[40] Fischer M (2017). Census and evaluation of p53 target genes. Oncogene.

[41] Su D, Wang X, Campbell MR, Song L, Safi A, Crawford GE (2015). Interactions of chromatin context, binding site sequence content, and sequence evolution in stress-induced p53 occupancy and transactivation. PLoS Genet.

[42] Sammons MA, Nguyen TT, McDade SS, Fischer M (2020). Tumor suppressor p53: from engaging DNA to target gene regulation. Nucleic Acids Res.

[43] Galindo-Moreno M, Giraldez S, Limon-Mortes MC, Belmonte-Fernandez A, Saez C, Japon MA (2020). p53 and FBXW7: sometimes two guardians are worse than one. Cancers.

[44] Leslie PL, Franklin DA, Liu Y, Zhang Y (2018). p53 regulates the expression of LRP1 and apoptosis through a stress intensity-dependent microRNA feedback loop. Cell Rep.

[45] Engeland K (2018). Cell cycle arrest through indirect transcriptional repression by p53: I have a DREAM. Cell Death Differ.

[46] Engeland K (2022). Cell cycle regulation: p53-p21-RB signaling. Cell Death Differ.

[47] Fischer M, Steiner L, Engeland K (2014). The transcription factor p53: not a repressor, solely an activator. Cell Cycle.

[48] Sullivan KD, Galbraith MD, Andrysik Z, Espinosa JM (2018). Mechanisms of transcriptional regulation by p53. Cell Death Differ.

[49] Baylin SB (2002). Mechanisms underlying epigenetically mediated gene silencing in cancer. Semin Cancer Biol.

[50] Simon JA, Kingston RE (2013). Occupying chromatin: Polycomb mechanisms for getting to genomic targets, stopping transcriptional traffic, and staying put. Mol Cell.

[51] Blackledge NP, Rose NR, Klose RJ (2015). Targeting Polycomb systems to regulate gene expression: modifications to a complex story. Nat Rev Mol Cell Biol.

[52] Cao R, Wang L, Wang H, Xia L, Erdjument-Bromage H, Tempst P (2002). Role of histone H3 lysine 27 methylation in Polycomb-group silencing. Science.

[53] Wang H, Wang L, Erdjument-Bromage H, Vidal M, Tempst P, Jones RS (2004). Role of histone H2A ubiquitination in Polycomb silencing. Nature.

[54] Andrysik Z, Bender H, Galbraith MD, Espinosa JM (2021). Multi-omics analysis reveals contextual tumor suppressive and oncogenic gene modules within the acute hypoxic response. Nat Commun.

[55] Subramanian A, Tamayo P, Mootha VK, Mukherjee S, Ebert BL, Gillette MA (2005). Gene set enrichment analysis: a knowledge-based approach for interpreting genome-wide expression profiles. Proc Natl Acad Sci USA.

[56] Qi W, Chan H, Teng L, Li L, Chuai S, Zhang R (2012). Selective inhibition of Ezh2 by a small molecule inhibitor blocks tumor cells proliferation. Proc Natl Acad Sci USA.

[57] Lue JK, Amengual JE (2018). Emerging EZH2 Inhibitors and their application in lymphoma. Curr Hematol Malig Rep.

[58] Gnyszka A, Jastrzebski Z, Flis S (2013). DNA methyltransferase inhibitors and their emerging role in epigenetic therapy of cancer. Anticancer Res.

[59] Riege K, Kretzmer H, Sahm A, McDade SS, Hoffmann S, Fischer M (2020). Dissecting the DNA binding landscape and gene regulatory network of p63 and p53. Elife.

[60] Wei JW, Huang K, Yang C, Kang CS (2017). Non-coding RNAs as regulators in epigenetics (Review). Oncol Rep.

[61] Andrysik Z, Kim J, Tan AC, Espinosa JM (2013). A genetic screen identifies TCF3/E2A and TRIAP1 as pathway-specific regulators of the cellular response to p53 activation. Cell Rep.

[62] Levrero M, De Laurenzi V, Costanzo A, Gong J, Melino G, Wang JY (1999). Structure, function, and regulation of p63 and p73. Cell Death Differ.

[63] Levrero M, De Laurenzi V, Costanzo A, Gong J, Wang JY, Melino G (2000). The p53/p63/p73 family of transcription factors: overlapping and distinct functions. J Cell Sci.

[64] Woodstock DL, Sammons MA, Fischer M (2021). p63 and p53: Collaborative partners or dueling rivals?. Front Cell Dev Biol.

[65] Visvader JE, Lindeman GJ (2012). Cancer stem cells: current status and evolving complexities. Cell Stem Cell.

[66] Clevers H (2011). The cancer stem cell: premises, promises, and challenges. Nat Med.

[67] Ye X, Tam WL, Shibue T, Kaygusuz Y, Reinhardt F, Ng Eaton E (2015). Distinct EMT programs control normal mammary stem cells and tumour-initiating cells. Nature.

[68] Teng YD, Wang L, Kabatas S, Ulrich H, Zafonte RD (2018). Cancer stem cells or tumor survival cells?. Stem Cells Dev.

[69] Yan M, Liu Q (2016). Differentiation therapy: a promising strategy for cancer treatment. Chin J Cancer.

[70] Hansen LA, Sigman CC, Andreola F, Ross SA, Kelloff GJ, De Luca LM (2000). Retinoids in chemoprevention and differentiation therapy. Carcinogenesis.

[71] Kogut I, McCarthy SM, Pavlova M, Astling DP, Chen X, Jakimenko A (2018). High-efficiency RNA-based reprogramming of human primary fibroblasts. Nat Commun.

[72] Hirai H, Tani T, Katoku-Kikyo N, Kellner S, Karian P, Firpo M (2011). Radical acceleration of nuclear reprogramming by chromatin remodeling with the transactivation domain of MyoD. Stem Cells.

[73] Tohyama S, Hattori F, Sano M, Hishiki T, Nagahata Y, Matsuura T (2013). Distinct metabolic flow enables large-scale purification of mouse and human pluripotent stem cell-derived cardiomyocytes. Cell Stem Cell.

[74] Shechter D, Dormann HL, Allis CD, Hake SB (2007). Extraction, purification and analysis of histones. Nat Protoc.

[75] Bushnell B, Rood J, Singer E (2017). BBMerge—Accurate paired shotgun read merging via overlap. PLoS One.

[76] Kim D, Paggi JM, Park C, Bennett C, Salzberg SL (2019). Graph-based genome alignment and genotyping with HISAT2 and HISAT-genotype. Nat Biotechnol.

[77] Li H, Handsaker B, Wysoker A, Fennell T, Ruan J, Homer N (2009). The sequence alignment/map format and SAMtools. Bioinformatics.

[78] Anders S, Pyl PT, Huber W (2015). HTSeq-a Python framework to work with high-throughput sequencing data. Bioinformatics.

[79] Love MI, Huber W, Anders S (2014). Moderated estimation of fold change and dispersion for RNA-seq data with DESeq2. Genome Biol.

