# Supplementary material for: Cell differentiation modifies the p53 transcriptional program through a combination of gene silencing and constitutive transactivation
Source: Cell Death Differ. 2023 Jan 21;30(4):952–65. doi: 10.1038/s41418-023-01113-4 (PMC10070495; doi:10.1038/s41418-023-01113-4)
Supplement: Supplementary file 1 — Supplemental Material [file 41418_2023_1113_MOESM1_ESM.docx]

**SUPPLEMENTAL MATERIALS.**


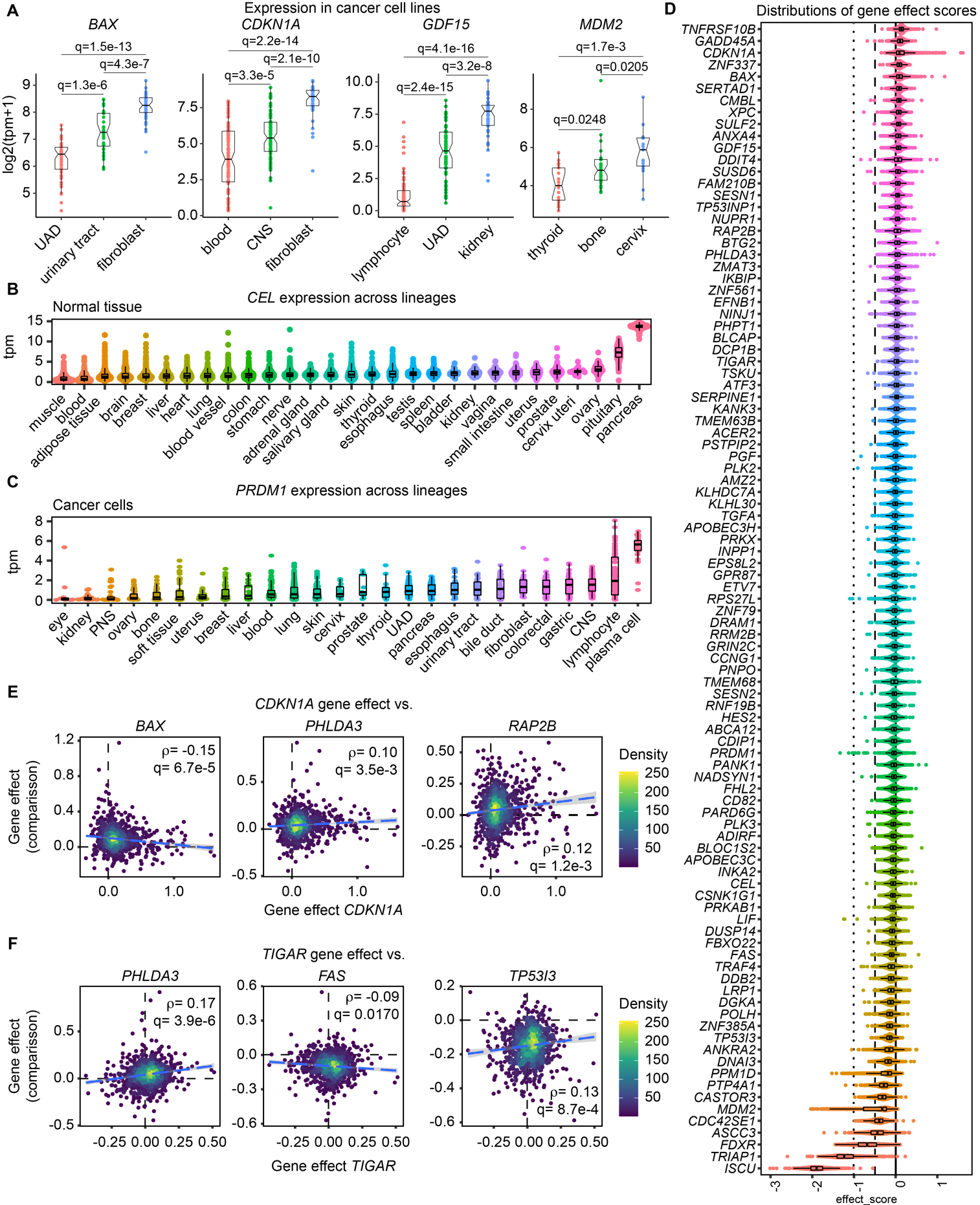
**Figure S1. Heterogeneity of the p53 transcriptional program across tissues and cancer cell types.**

(**A**) Sina plots displaying distributions of mRNA expression for *BAX, CDKN1A, GDF15*, and *MDM2* in cancer cell lineages with the lowest (red), median (green) and highest (blue) expression. TPM values were adjusted by log2(TPM+1). Sample sizes are as follows: upper aerodigestive (UAD, n= 56), urinary tract (n= 36), fibroblast (n= 39), blood (n= 103), central nervous system (CNS, n= 84), lymphocyte (n= 83), kidney (n= 38), thyroid (n= 17), bone (n= 39), cervix (n= 17). Significance was defined by Wilcox test with Benjamini Hochberg method for multiple hypothesis correction. Boxes indicate the median and interquartile ranges.

(**B**) Sina plots displaying distributions of mRNA expression for *CEL* across 29 normal tissue types. Sample sizes are as follows: muscle (n= 803), blood (n= 929), adipose tissue (n= 1204), brain (n= 2642), breast (n= 459), liver (n=226), heart (n= 861), lung (n= 578), blood vessel (n= 1335), colon (n= 779), stomach (n= 359), nerve (n= 619), adrenal gland (n= 258), salivary gland (n= 162), skin (n= 1809), thyroid (n= 653), esophagus (n= 1445), testis (n= 361), spleen (n= 241), bladder (n= 21), kidney (n= 89), vagina (n= 156), small intestine (n= 187), uterus (n= 142), prostate (n= 245), cervix uteri (n= 19), ovary (n= 180), pituitary (n= 283), pancreas (n= 328).

(**C**) Sina plots displaying distributions of mRNA expression for *PRDM1* across 26 cancer cell lineages. Sample sizes are as follows: eye (n= 11), kidney (n= 38), peripheral nervous system (PNS, n= 33), ovary (n= 64), bone (n= 39), soft tissue (n= 61), uterus (n= 40), breast (n= 61), liver (n= 24), blood (n= 103), lung (n= 207), skin (n= 84), cervix (n= 17), prostate (n= 11), thyroid (n= 17), upper aerodigestive (UAD, n= 56), pancreas (n= 52), esophagus (n= 32), urinary tract (n= 36), bile duct (n= 41), fibroblast (n= 39), colorectal (n= 71), gastric (n= 40), central nervous system (CNS, n= 84), lymphocyte (n= 83), plasma cell (n= 30). Boxes indicate the median and interquartile ranges.

(**D**) Sina plots displaying distributions of gene effect scores for 103 p53 core target genes across 1070 cancer cell lines. Dashed line (gene effect score = -0.5) represents essentiality for cell viability. Dotted line (gene effect score = -1.0) represents strong killing effect upon gene knock-out. Boxes indicate the median and interquartile ranges.

(**E**) Scatter plots showing gene effect of *CDKN1A* compared to *BAX* (left), *PHLDA3* (middle) and *RAP2B* (right) across 1070 cancer cell lines. Asterisks indicate significance by Spearman association test after multiple hypothesis correction using Benjamini Hochberg method (q < 0.1, FDR < 10%).

(**F**) Scatter plot showing gene effect of *TIGAR* compared to *PHLDA3* (left), *FAS* (middle) and *TP53I3* (right) across 1070 cancer cell lines. Asterisks indicate significance by Spearman association test after multiple hypothesis correction using Benjamini Hochberg method (q < 0.1, FDR < 10%).

**
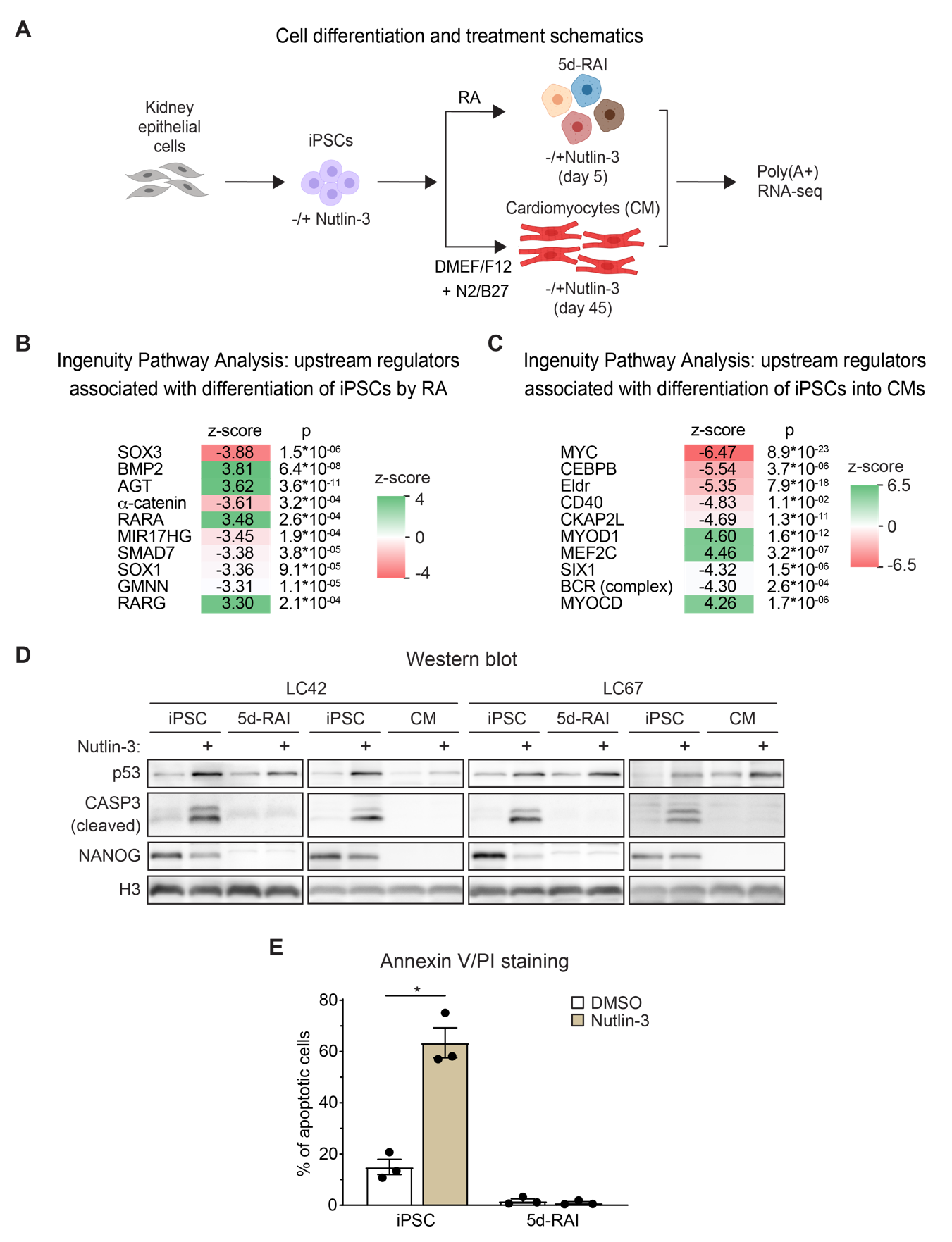
**

**Figure S2. Experimental paradigm for investigation of p53 action in different cellular states.**

**­**(**A**) Schematic representation of iPSC generation and differentiation via short-term retinoic acid treatment (5d-RAI) or into cardiomyocytes (CM).

(**B-C**) Ingenuity Pathway Analysis (IPA) of upstream regulators associated with iPSC differentiation with RA (**B**) or into CM (**C**). Top 2000 differentially expressed genes (by q<0.1 as defined by DESeq2) were used for the analysis. For full list of the predicted upstream regulators see Supplemental File 4. p values are calculated by IPA using a hypergeometric test.

(**D**) Immunoblots of cell lines LC42 and LC67 grown as iPSCs and differentiated into 5d-RAI cells or cardiomyocytes (CM) showing protein levels for p53, cleaved CASP3, the pluripotency marker NANOG, and the loading control histone H3.

(**E**) Annexin V-FITC/propidium iodide staining. Both iPSC and 5d-RAI cells were treated with 2.5 μM Nutlin-3 and vehicle (DMSO) for 12 hours. Harvested and stained cells were analyzed by flow cytometry.

**
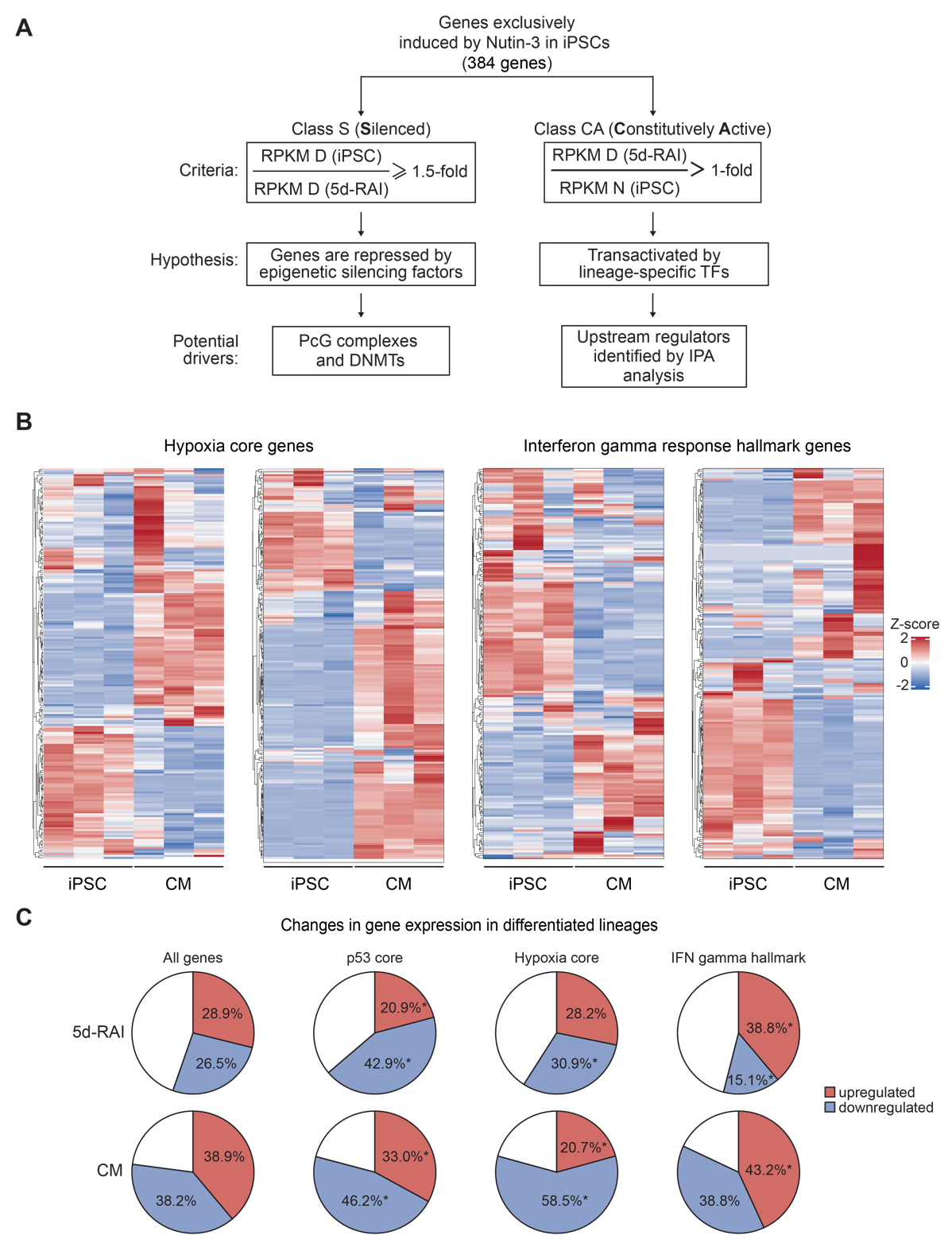
**

**Figure S3. Classification criteria for genes induced upon Nutlin treatment in different iPSCs versus differentiated cells.**

(**A**) Flowchart showing the stepwise approach for classification of gene sets, hypothesis generation, and identification of potential driving mechanisms. Class S indicates genes that are no longer induced because of gene silencing. Class CA indicates genes that are no longer induced because they are constitutively active in a differentiated cellular state.

(**B**) Heatmaps of expression level z-scores of hypoxia core response genes and interferon hallmark genes.

(**C**) Pie charts showing fractions of upregulated (red), downregulated (blue), and genes without a significant change in expression level (white) as iPSC differentiate upon RA treatment or into CM lineage. Changes across the transcriptome (all genes, n = 18062) were compared to p53 core target genes (n = 91), hypoxia core response genes (n = 188), and to interferon hallmark genes (n = 139) using hypergeometric test. Significant differences (p < 0.05) were marked with an asterisk.

**
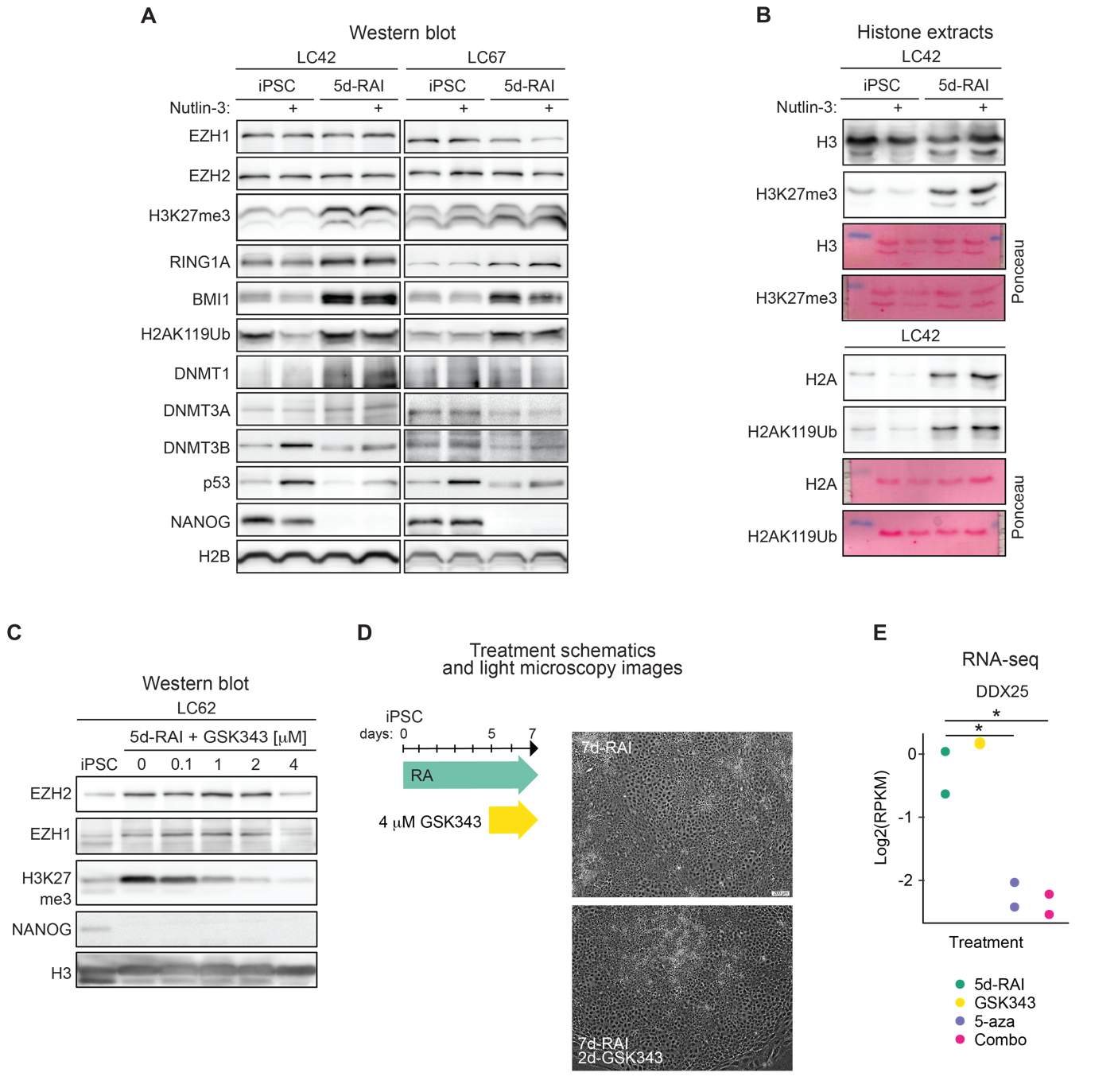
**

**Figure S4. Experimental design for investigation of epigenetic silencing of p53 target genes.**

(**A**) Immunoblots of potential regulators of Class S genes in the LC42 and LC67 cell lines, including subunits of the PRC2 complex (EZH1, EZH2), ­­histone H3 lysine 27 tri-methylation (H3K27me3), subunits of the PRC1 complex (RING1A, BMI1), histone H2A lysine 119 ubiquitination (H2AK119Ub), DNA methyltransferases (DNMT1, DNMT3A, and DNMT3B), p53, the pluripotency marker NANOG, and the loading control histone H2B.

(**B**) Western blots of histone acidic extraction of H3 / H3K27me3 and H2A / H2AK119Ub using the cell line LC42. Ponceau S stain was used for loading control.

(**C**) Immunoblot of 5d-RAI cells (line LC62) treated with increasing concentration of the EZH2 inhibitor GSK343 showing the levels of EZH1/2 and H3K27me3.

(**D**) Treatment schematics and phase contrast light microscopy of RA-differentiated lineage treated with 4 µM GSK343 as indicated. The scale bar is 0.2 mm long.

(**E**) Plot displaying gene expression (reads per kilobase per million, RPKM) for *DDX25*. An asterisk (*) denotes a statistically significant difference (p < 0.05) by DEseq2.

**
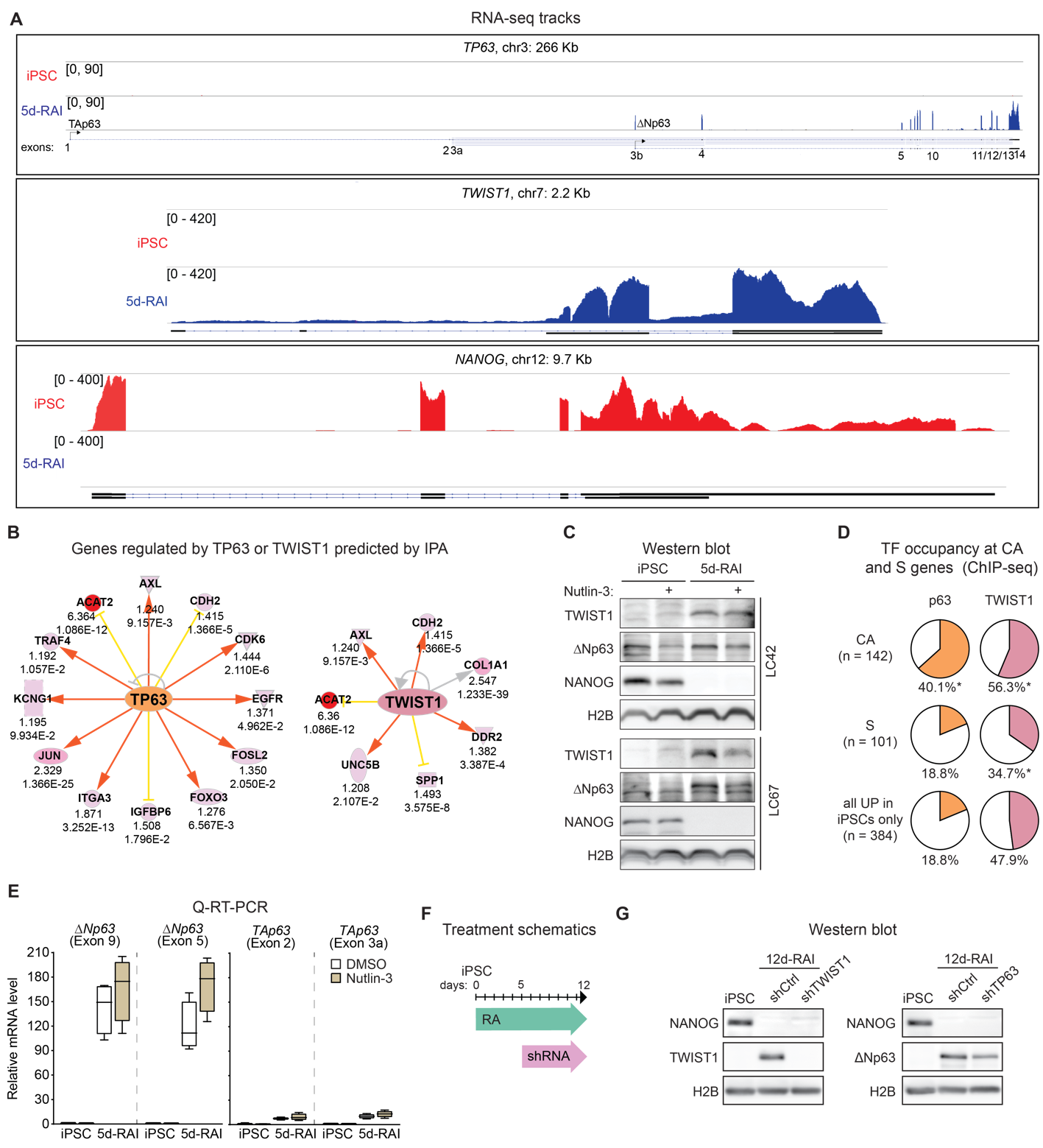
**

**Figure S5. Lineage-specific transcription factors transactivate a fraction of the p53 network upon cell differentiation.**

(**A**) Genome track views displaying the mapped RNA-seq reads for *TP63*, *TWIST1* and *NANOG* in the cell line LC62 (iPSC versus 5d-RAI). The *TP63* gene is transcribed from exon 3b yielding the *∆N*p*63* isoform in the 5d-RAI differentiated state. NANOG is used to illustrate the impact of cell differentiation.

(**B**) Ingenuity pathway analysis (IPA) gene networks for TWIST1 and TP63 in 5d-RAI cells.

(**C**) Western blot of cell lines LC42 and LC67 (iPSC and 5d-RAI) showing increased expression of TWIST1 and ∆Np63 upon cell differentiation

(**D**) Occupancy of transcription factors obtained from published data p63 and TWIST1 within 2.5 kb from transcription start sites of CA genes. An asterisk denotes p < 0.05 as determined by hypergeometric test in comparison to “all UP in iPSC only” group.

(**E**) Results of isoform-specific Q-RT-PCR analysis for two major isoforms of *TP63* (*TAp63 versus ∆N*p*63*) showing increased expression and induction of *∆N*p*63* in 5d-RAI isogenic lineages. Data are presented as SEM (n = 4). An asterisk denotes p < 0.05 (t-test).

(**F**) Treatment schematics applicable to (**G**).

(**G**) Western blots of RA-differentiated cells depleted of TWIST1 or p63, respectively.
