# Supplementary material for: Cell differentiation modifies the p53 transcriptional program through a combination of gene silencing and constitutive transactivation
Source: Cell Death Differ. 2023 Jan 21;30(4):952–65. doi: 10.1038/s41418-023-01113-4 (PMC10070495; doi:10.1038/s41418-023-01113-4)
Supplement: Supplementary file 10 — Original Data Files [file 41418_2023_1113_MOESM10_ESM.pdf]

Supplemental Files Legends:

Supplemental Table 1 - shRNA sequences. Microsoft Excel spreadsheet.

Supplemental Table 2 - Primers used for Q-RT-PCR. Microsoft Excel spreadsheet.

Supplemental Table 3 - Primary and secondary antibodies. Microsoft Excel spreadsheet.

Supplemental File 1 - p53 target gene expression data in GTEx and DepMap (related to Figure 1). Microsoft Excel spreadsheet.

Supplemental File 2 - DepMap genetic co-dependency data (related to Figure 1). Microsoft Excel spreadsheet.

Supplemental File 3 - RNA-seq DESeq2 output (related to Figure 2). Microsoft Excel spreadsheet.

Supplemental File 4 - Ingenuity Pathway Analysis data (related to Figure 2). Microsoft Excel spreadsheet.

Supplemental File 5 - Class S RNA-seq DEGs (related to Figure 4). Microsoft Excel spreadsheet.

Supplemental File 6 - Original Data Files. Uncropped Western blots. Adobe PDF file.
